# Mechanism and design of allosteric activators of SIRT1

**DOI:** 10.1093/procel/pwac039

**Published:** 2022-09-03

**Authors:** Fei Liu, Ningning Pang, Rui-Ming Xu, Na Yang

**Affiliations:** State Key Laboratory of Medicinal Chemical Biology, College of Pharmacy and Key Laboratory of Medical Data Analysis and Statistical Research of Tianjin, Nankai University, Tianjin 300353, China; State Key Laboratory of Medicinal Chemical Biology, College of Pharmacy and Key Laboratory of Medical Data Analysis and Statistical Research of Tianjin, Nankai University, Tianjin 300353, China; National Laboratory of Biomacromolecules, CAS Center for Excellence in Biomacromolecules, Institute of Biophysics, Chinese Academy of Sciences, Beijing 100101, China; State Key Laboratory of Medicinal Chemical Biology, College of Pharmacy and Key Laboratory of Medical Data Analysis and Statistical Research of Tianjin, Nankai University, Tianjin 300353, China

**Dear Editor,**

The sirtuin family Nicotinamide adenine dinucleotide-dependent deacetylases have important roles in many biological processes (Milne and Denu, 2008). SIRT1 is a mammalian homolog of yeast Sir2, which is the founding member of the sirtuin family. Many SIRT1 substrates have been reported, including histones, p53, FOXOs, and PGC1α (Hsu et al., 2013). Stimulating the activity of SIRT1 has been shown to be an attractive therapeutic strategy for various physiological and pathological conditions such as aging, metabolic disorders, inflammation, and neurodegeneration (Guarente, 2011). Several small molecules have been identified or developed as potential sirtuin-activating compounds (STACs). Most notably, resveratrol, a natural STAC, was shown to activate the SIRT1-catalyzed deacetylation of the Fluor de Lys (FDL) peptide, which is a p53 peptide fused to a 7-amino-4-methylcoumarin (AMC) fluorophore at the C-terminus (Table S1; Howitz et al., 2003). However, the activation of deacetylation of the p53 peptide is dependent on the presence of the AMC fluorophore (Borra et al., 2005; Wu et al., 2013). Compared to resveratrol, synthetic STACs SRT1720, SRT2183, and SRT1460 were reported to activate human SIRT1 much more potently (Milne et al., 2007). However, subsequent experiments with these three synthetic STACs and resveratrol, using substrates including wild-type p53 and a p53 peptide labeled with 5-carboxytetramethylrhodamine (TAMRA) at the acetylated lysine, revealed that the chemical modification groups were necessary for SIRT1 activation, as no activating effects were observed with the unmodified substrate (Pacholec et al., 2010). Dai et al. argued that the TAMRA label did not directly interact with the STACs, but promoted allosteric activation of SIRT1 (Dai et al., 2010). Assisted allosteric activation was also reported when SIRT1 catalyzed the deacetylation of native substrates PGC1α and FOXO3a, which contain hydrophobic residues at the + 1 or  + 6 positions relative to the acetylated lysine. They proposed that the side chains of these hydrophobic residues mimic the function of AMC or TAMRA labels during the activation of SIRT1 (Hubbard et al., 2013), but the results are controversial (Cao et al., 2015).

We previously reported on the structure of the activated SIRT1-FDL-resveratrol complex in a closed conformation (Cao et al., 2015). The SIRT1 N-terminal domain (NTD) was close to the catalytic domain (CD), and resveratrol and NTD synergistically increased the binding affinity of FDL to SIRT1. We also revealed that the AMC group in the FDL peptide would occupy the path of the main-chain position of a native substrate, thus the side chain of the  + 1 or  + 6 residue of the substrate cannot bind SIRT1 in a way similar to that of the AMC group (Cao et al., 2015). Another study reported the structures of SIRT1 complexes involving ac1 (a synthetic STAC, SRT1720 derivative) and Cp53 substrate in an open conformation (Table S1), showing that the NTD was located far away from the CD and ac1 only interacted with NTD (Dai et al., 2015). The binding of ac1 to NTD was found to regulate allosteric activation of SIRT1, based on studies using hydrogen–deuterium exchange mass spectrometry (HDXMS) (Dai et al., 2015). However, we found the SIRT1-ac1 complex with an open conformation of NTD-lacked structural and thermodynamic stability (Liu and Yang, 2020). And the questions with regard to whether the open conformation is an important state during the assisted allosteric activation of SIRT1, and how could we quantify the conformational space involved in the allosteric activation of SIRT1 and identify the relationship correlating the conformational space and the biochemical results remain open. To address these questions, molecular dynamics simulations can serve as a powerful tool to provide more insights and quantitative information regarding underlying biological mechanisms (Whitford et al., 2007; Lu and Wang, 2008). Here, we developed a series of molecular dynamic simulation models to explore the mechanism involved in assisted allosteric activation of SIRT1 (Fig. S1). Based on the activation mechanism revealed by the simulations and experimental data, a high-throughput molecular docking (HMD) strategy was developed and several compounds activating the SIRT1-catalyzed deacetylation were identified and verified.

To investigate allosteric dynamic pathways of SIRT1 involving the STAC ac1, we performed all-atom molecular dynamic (AAMD) simulations of the SIRT1-Cp53-ac1 system (Figs. 1A and S2). The initial conformation involved an open NTD conformation (Protein Data Bank [PDB] ID: 4ZZJ). SIRT1 NTD was about 4.3 nm (centroid distance) from SIRT1 CD, and activator ac1 tended to only interact with NTD (not with substrate Cp53 or SIRT1 CD). After monitoring the SIRT1 root-mean-square deviation (RMSD) values relative to the closed SIRT1 conformation (PDB ID: 5BTR) (Figs. 1A, S2A, and S2B), and the centroid distances between ac1 and the substrate’s acetylated lysine (Fig. S2C), as well as between ac1 and NTD (Fig. S2D), we identified two pathways regarding the conformational changes in this system. In pathway 1, NTD interacted with CD and Cp53, but ac1 did not interact with Cp53 or CD in the final dynamic states (Fig. S2B and S2E). In pathway 2, E230 of NTD and R446 of CD formed interaction with each other, and ac1 interacted with Cp53 after 50 ns of simulations, and the state was stable thereafter (Fig. S2B and S2E). Moreover, we assessed 10 independent 70-ns trajectory simulations, trj1-trj10 (Figs. 1A and S2F). A SIRT1-Cp53-ac1 conformation similar to the final stable conformation of pathway 1 was formed with frequency in five of these simulations (trj1, trj2, trj3, trj6, and trj8). By contrast, a conformation similar to the final stable conformation of pathway 2 was formed in two of these simulations (trj5 and trj9) (Fig. 1A). In the remaining three simulations (trj4, trj7, and trj10), NTD did not interact with Cp53 (Fig. S2F). Based on the RMSD values of Cp53 position relative to the initial open structure and the distance between ac1 and NTD, Cp53 and ac1 underwent smaller structural fluctuation in pathway 2 than did in pathway 1 (Fig. S2B and S2D), indicating more stable conformations of SIRT1-Cp53-ac1 were formed in pathway 2.

**Figure 1.**
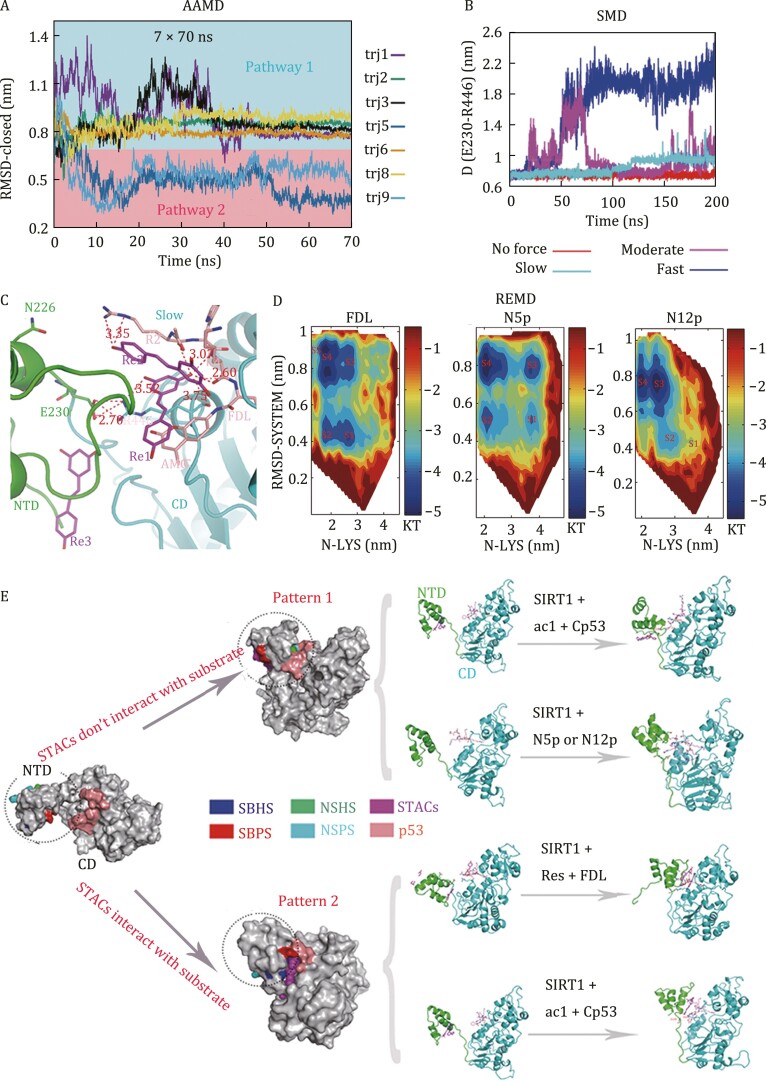
Mechanism of allosteric activation of SIRT1. (A) Seven 70-ns trajectories (trj) of RMSD-closed for the SIRT1-Cp53-ac1 system. RMSD-closed is the SIRT1 root-mean-square deviation (RMSD) values relative to the closed SIRT1 conformation (PDB ID: 5BTR). Although 10 independent 70-ns trj were performed, only seven (trj1, 2, 3, 5, 6, 8, and 9) are shown because SIRT1 NTD did not interact with Cp53 in the other three (trj4, trj7, or trj10). (B) Dynamic trajectories of the SIRT1-FDL-resveratrol system with AAMD (red: no force) and SMD (cyan: slow pulling rate, magenta: moderate pulling rate, blue: fast pulling rate). D (E230-R446) are the centroid distances between polar residues E230 and R446 of SIRT1. (C) Structures extracted from the slow pulling SMD simulations. Salmon: p53 peptide; magenta: resveratrols; green: SIRT1 NTD; cyan: SIRT1 CD. (D) Free energy landscapes of the SIRT1-FDL, SIRT1-N5p, and SIRT1-N12p systems. *Y*-axes show RMSD-SYSTEM, which are the RMSD values of SIRT1 and p53 relative to the initial structure. X axes show N-LYS, which is the centroid distance between NTD and the substrate’s acetylated lysine. Free energy surfaces are colored as gradients (units are kT). Thermodynamic states (FDL-s1 to FDL-s5, N5p-s1 to N5p-s4, and N12p-s1 to N12p-s4) are marked in each landscape. (E) A model of allosteric activation of SIRT1. In pattern 1, the STAC does not interact with the p53 substrate. In pattern 2, the STAC interacts with the p53 substrate. Magenta: STAC; salmon: p53 peptide; dotted circle: SIRT1 NTD. NSHS: Non-STAC-binding hydrophobic site; NSPS: Non-STAC-binding polar site; SBHS: STAC-binding hydrophobic site; SBPS: STAC-binding polar site.

Then we performed one AAMD simulation (no force) and three steered molecular dynamic (SMD) simulations (with slow, moderate, and fast pulling rates, respectively) of the closed structure of SIRT1-FDL-resveratrol (PDB ID: 5BTR) to explore the dynamic pathway of the opening of the closed NTD Conformation (Figs. 1B and S3). Based on the AAMD simulations, monitoring RMSD values relative to the closed SIRT1 conformation (RMSD-closed) and the centroid distances between R2 of FDL and polar residues Q222 and N226 of SIRT1 (D(R2-PS)) revealed that the closed conformation was very stable and had few dynamic fluctuations (red trajectories in Figs. 1B and S3A). Next, to monitor the opening of the closed NTD conformation in the presence of resveratrol (Res1, Res2, and Res3) across a finite simulation time scale, three SMD simulations were performed with a pulling force exerted on NTD. The opening of the closed NTD conformation depended on resveratrol-mediated regulation of (i) the NTD-CD interactions between E230 and R446, and (ii) the FDL-NTD interactions (Fig. 1C). In the slow pulling simulations, the E230-R446 interaction (D(E230-R446)) did not break during the entire 200 ns of simulations (cyan trajectory in Fig. 1B). Regarding the moderate pulling simulations, this interaction exhibited a dynamic balance between formation and fracture (magenta trajectory in Fig. 1B). And in the fast pulling simulations, this interaction was broken after 50 ns of the pulling simulations and could not be formed again (blue trajectory in Fig. 1B). Furthermore, in the slow pulling simulations (Fig. 1C), as the E230-R446 interaction continued, the FDL (R2) -NTD (N226) interaction was broken; Res2 continued to interact with R2 but the interactions between Res2 and NTD residues Q222 and N226 were broken. In contrast, regarding the moderate and fast pulling simulations, when the E230-R446 interaction was broken, Res2 continued to interact with Q222 of NTD (Fig. S3B and S3C). These observations indicated that Res2 regulated the FDL (R2)-NTD (Q222 and N226) interactions along with the formation and breakage of the E230-R446 interaction. For all three pulling simulations, Res1 continued interacting with FDL (AMC group), although the distance between NTD and CD was further than 4.3 nm (Fig. S3C). This suggested that Res1 interacted with FDL (AMC group) before the formation of the E230-R446 interaction. Regarding Res3, we did not find evidence that it can regulate the NTD-FDL interaction by SMD simulations. In summary, we found a dynamic pathway of the opening of the closed NTD conformation depended on Res1/Res2-mediated regulation of the interactions between E230-R446 and FDL-NTD.

Furthermore, we used replica exchange molecular dynamic (REMD) simulations to explore the interactions between SIRT1 and different substrates without STACs. First, we constructed free energy landscapes for the scenarios of SIRT1 interacting with three p53 peptides (FDL, N5p, and N12p) (Fig. 1D; Table S1). The RMSD values relative to the initial structure (designated “RMSD-SYSTEM”) were used to monitor structural changes of SIRT1-p53 during the simulations (Fig. 1D). The centroid distance between NTD and the substrate’s acetylated lysine (designated “N-LYS”) was used to assess the movement of NTD. Regarding the landscape of the SIRT1-FDL system, there were five stable states when the N-LYS values were less than 3.0 nm. Regarding the landscape of the SIRT1-N5p system, there were four stable states, and all of their N-LYS values were less than 3.7 nm. For the landscape of the SIRT1-N12p system, the N-LYS values of two stable states (s3 and s4) were less than 2.5 nm. The N-LYS values of all states in all three systems were lower than the centroid distance between NTD and CD in the open NTD conformation (4.3 nm), among which states s4 and s5 exhibited the lowest N-LYS values in each system. Comparing the structures of the different states, NTD was near CD and the p53 substrate in all s4 states (FDL-s4, N5p-s4, and N12p-s4) and FDL-s5, while NTD was further away from CD in all s1 states (FDL-s1, N5p-s1, and N12p-s1) (Fig. S4). The thermodynamic states of all s4 and FDL-s5 represented a conformation that NTD interacted with p53 substrate, and other thermodynamic states (s1, s2, and s3) showed intermediate conformations of NTD moving to p53. As the N-LYS decreased, the thermodynamic states began to be stable, which indicated that SIRT1 underwent a more stable conformation when NTD was near the substrate. In fact, the exact thermodynamic pathways of the conformational change of SIRT1 were depended on the type of p53 peptide. The SIRT1 systems with shorter peptides (FDL and N5p) had two-pathway landscapes (Fig. 1D). For the FDL s1-s2-s4-s5 and N5p s1-s2-s4 pathways, the conformational change of SIRT1-p53 system (RMSD-SYSTEM) occurred after NTD began to move near CD (N-LYS). Regarding the FDL s1-s3-s4-s5 and N5p s1-s3-s4 pathways, the conformational change of SIRT1-p53 system began to occur before the movement of NTD to CD (N-LYS). In contrast, in the free energy landscape of the SIRT1-N12p system, there was only one pathway from s1 to s4 (s1-s2-s3-s4), and the increase of RMSD-SYSTEM is more relevant to the decrease of N-LYS from state s2 to s3 (Fig. 1D). Monitoring the contact map and structure of each thermodynamic state showed that in the FDL-s1, FDL-s2, and N12p-s2 states (intermediate states in which NTD moved toward CD and the p53 peptides), the E230-R446 interactions were already formed (Figs. S4–8). This phenomenon indicated that the E230-R446 interactions might be formed before NTD interacts with p53, thus promoting the NTD movement without STACs. In the states of FDL-s5, N5p-s2, N5p-s4, N12p-s3, and N12p-s4 (in which NTD interacted with the p53 peptides), there were various interaction sites between NTD and p53 (Figs. S9–11).

We divided these substrate-binding sites in NTD into the following four classes (Fig. 1E): (i) STAC-binding hydrophobic site (SBHS), as exampled in the FDL-s5 state, where the AMC group of FDL directly interacted with the ac1-binding hydrophobic residues of NTD, including P212, I223, and I227 (Fig. S9). (ii) STAC-binding polar site (SBPS), as shown in the N12p-s3 state, where arginine (−3) of N12p interacted with the resveratrol-binding polar residues of NTD, including N226 and Q222 (Fig. S10). (iii) Non-STAC-binding hydrophobic site (NSHS), as in the N12p-s4 state, where phenylalanine (+6) of N12p interacted with the hydrophobic L228 of NTD, while in the N5p-s2 and N5p-s4 states, leucine (+1) of N5p interacted with the hydrophobic F187 of NTD (both L228 and F187 are not reported STAC-binding sites) (Figs. S10 and S11). (iv) Non-STAC-binding polar site (NSPS), as in the N5p-s2 and N5p-s4 states, where arginine (−3) of N5p interacted with the polar sites around D204 of NTD (not a reported STAC-binding site) (Fig. S11).

The above results showed that thermodynamic pathways and conformational space of the SIRT1-p53 interaction varied widely depending on the type of p53 substrate. There have been many experimental studies concerning the activation of SIRT1, including the influence of different substrates or STACs (Howitz et al., 2003; Borra et al., 2005; Milne et al., 2007; Dai et al., 2010; Pacholec et al., 2010; Hubbard et al., 2013). However, the experimental data regarding the conformational space involved in the SIRT1-p53-STAC interactions are lacking. To fill this gap, we then examined *in vitro* deacetylation activities of SIRT1 against various p53 substrates by enzyme-coupled deacetylation (ECD) assays. The deacetylation rates were assessed in the absence or presence of STACs, resveratrol or SRT1720 (Fig. 2A). When no STAC was present, the fastest deacetylation rate occurred for N12p peptide. It should be noted that resveratrol (100 μmol/L) and SRT1720 (100 μmol/L) accelerated the deacetylation rate on FDL peptide by about 3.5- and 2.3-fold, respectively. N5p deacetylation was not accelerated by resveratrol, and only about 1.3-fold by SRT1720 (100 μmol/L). N12p deacetylation was not accelerated by either resveratrol or SRT1720 (Fig. 2A). The results may be explained by our simulation results because the closed conformation of SIRT1-N12p was the easiest to form from the open conformation among three SIRT1-p53 systems among the simulations of SIRT1 on FDL and N5p substrate (Fig. 1D). To further test this assumption, we constructed a one-dimensional free energy landscape of the RMSD value of SIRT1-p53 without STACs to quantify the free energy barrier between open and closed conformation of SIRT1-p53 systems (Fig. S12). As RMSD increased, the landscape depicted the thermodynamic pathway regarding the interactions of NTD, p53, and CD to form a closed conformation. The SIRT1-N12p landscape exhibited many basins with lower barriers compared to the SIRT1-FDL and SIRT1-N5p landscapes. Thus, without STACs, the barrier between thermodynamic states was easier to cross in the SIRT1-N12p landscape than in other landscapes.

**Figure 2.**
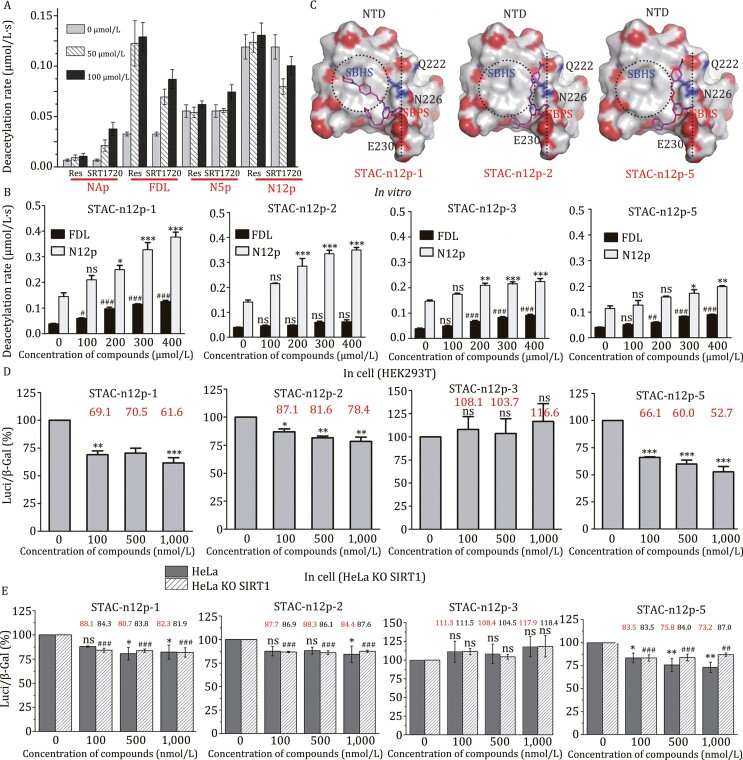
The design of allosteric activators of SIRT1. (A) *In vitro* enzyme-coupled deacetylation (ECD) assay results of SIRT1 against four kinds of p53 peptides in the presence of various concentrations of reported STACs resveratrol and SRT1720. The *X*-axis indicates the concentrations of compounds, and the *Y*-axis indicates the deacetylation rate of SIRT1 against substrate peptides. Sequences of these peptides are shown in Table S1. (B) ECD assay results of SIRT1 against FDL and N12p in the presence of various concentrations of newfound compounds. (C) Surface-binding model of NTD with newfound compounds including STAC-n12p-1, -2, and -5. The compounds are shown in a stick model in magenta. O, N, and C atoms in SIRT1 NTD are colored red, blue and gray, respectively. The SBHS is marked by a dotted circle and the SBPS is marked by a dotted line. (D and E) *In cell* activations of SIRT1 by newfound STACs examined by deacetylation assays in HEK 293T cells and in HeLa and HeLa KO SIRT1 cells, respectively. The *X*-axis indicates the concentrations of compounds, and the *Y*-axis shows reduction of luciferase activity indicating the activation activity of STACs. Data are mean ± SD. ^*^P or ^#^*P *< 0.05, ^**^P or ^##^*P* < 0.01, ^***^P or ^###^*P *< 0.001, ns indicates no statistical difference. Data are analyzed by one-way ANOVA.

Based on the above simulations, several insights into the mechanisms of SIRT1 allosteric activation can be gained. The simulations showed that the conformational changes of SIRT1-p53-STAC may be divided into two patterns, one in which the STAC does not interact with the substrate p53 and the other does (Fig. 1E). In pattern 1 where STACs don’t interact with the substrate, p53 interacts with the NSHS and NSPS of NTD, and the STAC binds to the SBHS and SBPS but it does not directly interact with p53 (Fig. 1E). This pattern can be observed by both AAMD and REMD: (i) The AAMD showed STAC ac1 induced the conformational change of SIRT1-Cp53 via pattern 1 (pathway 1 in Fig. 1A). (ii) Although it is hard to mimic the closed conformation of SIRT1-N5p-STAC and SIRT1-N12p-STAC, as knowledge about their open conformation is lacking, REMD simulations indicated that SIRT1-N5p and SIRT1-N12p might form pattern 1 closed conformations without STACs (Figs. S4, S10, and S11). Thus, binding of STAC to the SBHS and SBPS after the closed conformations of SIRT1-N12p or SIRT1-N5p are formed would represent the pattern 1 mechanism. In pattern 2 where STACs interact with the substrate, p53 interacts with the SBHS and SBPS of NTD, as well as the STACs (Fig. 1E). This pattern was observed by both SMD and REMD: (i) The SMD simulations indicated that the conformational change of SIRT1-FDL-resveratrol formed via the pattern 2 (Figs. 1B and S3). (ii) ac1 also induced the conformational change of SIRT1-Cp53 by pattern 2 (pathway 2 in Fig. 1A). Thus, according to the proposed patterns 1 and 2, there may be more than one mechanism underlying the allosteric activation of SIRT1. However, both the simulations and experimental evidences suggested that pattern 2 may be most likely for SIRT1-catalyzed deacetylation. First, according to the simulation, the open state of SIRT1-Cp53-ac1 could be transformed into a closed state by both pattern 1 and pattern 2 mechanisms. However, the Cp53 adopted a more stable conformation in the final closed state of pattern 2 mechanism, which may be beneficial to activating deacetylation of SIRT1. Second, ECD results showed that resveratrol and SRT1720 could not activate SIRT1 on wild-type substrates N5p or N12p. The observation that SIRT1 could be highly stimulated by resveratrol toward FDL is consistent with our simulation result that the closed conformation of SIRT1-FDL-resveratrol obeyed pattern 2 (Cao et al., 2015).

Based on the above results, we proposed that an ideal STAC of SIRT1 may meet two criteria: (i) STAC should bind to the hydrophobic SBHS surface before NTD moves close to the substrate in order to regulate the hydrophobic interaction between the SBHS and the substrates (Fig. S13A), and (ii) STAC should have a fragment that can simultaneously regulate the polar interaction between the SBPS and the substrate. Like in the SIRT1-FDL-resveratrol system (Fig. S13B), resveratrol activated the deacetylation of SIRT1 on FDL peptide efficiently because it met the two criteria above, but an authentic activator of SIRT1 for deacetylation of wild-type substrates still needs to be identified. To achieve this goal, we developed the HMD method to search the virtual compound library. A modified closed structure was used for HMD by replacing AMC group with a leucine and removing all resveratrols in the SIRT1-FDL-resveratrol model (Fig. S13C). The docking grid was centered on the geometric center of the leucine (Fig. S13C). The diameter of docking grid was set to about 1.8 nm. The grid was large enough to cover the potential binding region between NTD and CD. Finally, 20 compounds with the lowest binding free energy (Autodock_energy) were selected for further experimental examinations (Table S2).

*In vitro* ECD assays were first carried out to test the activities of these 20 compounds for catalytic activation of SIRT1 toward the wild-type substrate N12p and the fluorescent substrate FDL. Eight compounds were found to activate SIRT1 activity on N12p or on both N12p and FDL substrates (Figs. 2B, S14, and S15). Among these, STAC-n12p-1 and STAC-n12p-2 activated N12p deacetylation to the most extent. Other compounds (STAC-n12p-3 to STAC-n12p-8) had relatively less effect on the activity of SIRT1 against N12p. STAC-n12p-2 appeared to be an ideal activator as it accelerated the SIRT1 reaction only toward the N12p substrate, while STAC-n12p-1 activated deacetylation of both N12p and FDL. By contrast, STAC-n12p-7 activated FDL deacetylation well but the effect on N12p is barely satisfactory (Fig. S15). Although these eight compounds can activate SIRT1 activity on N12p, it is hard to predict the closed binding pattern of SRIT1-n12p-STAC without crystal structure. However, potential binding sites of STAC are located at NTD in the open conformation. We can predict the binding pattern of the eight compounds to NTD in the open conformation. The docking grid was centered on the geometric center of NTD after removing ac1 (Fig. S13A), and it was large enough to cover the potential binding surface on NTD. Comparing the docking pattern of these compounds to SIRT1-NTD, the nitrogen heterocyclic rings of STAC-n12p-1 and 2 bound to the SBPS region of the NTD between N226 and E230 by polar interactions (Fig. 2C). This region represents the N-terminus-binding sites of N12p in the absence of STACs. Thus, both compounds may be able to regulate the polar interaction between N-terminus of N12p and the SBPS sites of SIRT1-NTD. However, the other parts of the two compounds are bound to different sites of NTD. STAC-n12p-1 mainly interacted with the hydrophobic SBHS sites, while STAC-N12p-2 interacted with the polar SBPS sites. This provided a good explanation of why STAC-n12p-2 exhibited a good selection of activation on N12p but not on FDL deacetylation, as it cannot simultaneously regulate the hydrophobic interaction between FDL-AMC group and the SBHS sites of NTD. However, in the case of STAC-n12p-7, it interacted mostly with the hydrophobic SBHS surface of NTD, leading to efficient activation of deacetylation of FDL only (Fig. S16). To test the activities of the above newfound STACs on SIRT1 activation to other reported substrates, longer peptides of PGC1α and FOXO3a (Table S1) were used instead of p53 in the *in vitro* ECD assays. Results showed a rather strong activation of SIRT1 to PGC1α by STAC-n12p-1, 2, 3, 5, and considerable activation of SIRT1 to FOXO3a by STAC-n12p-1, 2, 3 (Fig. S17). These results indicate the newfound STACs may have the potential to activate SIRT1 deacetylation on general substrates.

Above we have identified a series of compounds (STAC-n12p-1 to STAC-n12p-8) that can activate SIRT1 activity on wild-type substrate peptides *in vitro*. Next, *in cell* activities of these compounds were examined by deacetylation assays of SIRT1 in HEK 293T cells (Figs. 2D and S18). From the results, we found compounds STAC-n12p-1 and STAC-n12p-5 showed better activation activity than resveratrol as the averaged Luci/β-Gal value was lowered to 69.1% and 66.1%, respectively, compared to those of 85.7% and 82.1% of resveratrol and SRT1720 at 100 nmol/L compound concentration. By contrast, STAC-n12p-2, 6, 8 showed similar activation activities as resveratrol, and STAC-n12p-3, 4, 7 had no *in cell* activity at all (Figs. 2D and S18). Similar deacetylation activities of SIRT1 activated by selected STACs were tested in SIRT1 knockout HeLa cells to remove endogenous interferences (Fig. 2E), which showed consistent activation results of STAC-n12p-1 and STAC-n12p-5. It is worth noting that STAC-n12p-1 has a high activation activity both *in vitro* and *in cell*. However, STAC-n12p-2, 3 activate SIRT1 less *in cell* though the *in vitro* activities were pretty good (Fig. 2D and 2E). This may be caused by their structural formula, as the rather high solubility in water caused difficulties to cross cell membranes (solubility trial [in water]: STAC-n12p-2- > STAC-n12p-3 > STAC-n12p-1 > STAC-n12p-5) (Fig. S14). Furthermore, we found the STAC-n12p-5 had similar activation activity as STAC-n12p-1 at 100 nmol/L *in cell*, but its activation activity was not remarkable *in vitro*. Comparing the docking patterns of STAC-n12p-5 to SIRT1 (Fig. 2C), the STAC-n12p-5 could also regulate the polar region between N226 and E230 on NTD as STAC-n12p-1 and STAC-n12p-2 did. But the low solubility in water of STAC-n12p-5 might hamper its *in vitro* activity.

In conclusion, here we have reported the global dynamic and thermodynamic landscapes of SIRT1 allosteric activation, which agree well with the experimental data. We found that there are more than one mechanism governing the allosteric activation of SIRT1, depending on the interacting mode between STACs and the substrates. Based on the two criteria for the high-efficiency activation mechanism of SIRT1 revealed by our simulations and experimental data, a high-throughput molecular docking strategy was developed and several compounds activating the SIRT1-catalyzed deacetylation were identified. *In vitro* and *in cell* activities of these newfound STACs were examined and the possible regulating mechanisms were analyzed. These analyses provided important clues for future iterative structure and activity relationship (SAR) studies. Future *in vivo* studies such as improvement of health and longevity of high-fat diet mice by these STACs through SIRT1 activation may help to pave their way to pre-clinical trials. In summary, our results provide sound evidence regarding the allosteric activation of SIRT1 and should facilitate the development of authentic STACs of SIRT1 for the benefit of human health.

## Supplementary Material

pwac039_suppl_Supplementary_Material

## Footnotes

We thank Dr. Sheng Wang at Huazhong University of Science and Technology for his advice on virtual screening. We thank Dr. Lu-Yuan Li at Nankai University and Dr. Ye Xiang at Tsinghua University for generous gift of HEK 293T cells and pcDNA5 plasmid. The molecular simulations were carried out at the National Supercomputer Center in Tianjin, and the calculations were performed on TianHe-1 (A).

This work was supported by the Chinese Ministry of Science and Technology, Natural Science Foundation of China (Grant Nos. 2018YFA0107004, 2019YFA0508900, 91853204, 31870737, 32170549 and 22103040), the National Postdoctoral Program for Innovative Talents (BX20180149), Haihe Laboratory of Cell Ecosystem Innovation Fund (HH22KYZX0021) and the State Key Laboratory of Medicinal Chemical Biology.

Na Yang and Rui-Ming Xu designed the study. Fei Liu designed and performed the computational studies. Ningning Pang performed the experimental studies. Fei Liu, Ningning Pang, Rui-Ming Xu and Na Yang wrote the manuscript. The authors declare no conflict of interest. Methods, including statements of data availability are available in the supplementary materials.
