# Supplementary material for: Mechanism and design of allosteric activators of SIRT1
Source: Protein Cell. 2022 Sep 3;14(5):387–92. doi: 10.1093/procel/pwac039 (PMC10166154; doi:10.1093/procel/pwac039)
Supplement: pwac039_suppl_Supplementary_Material [file pwac039_suppl_supplementary_material.doc]

**SI appendix**

**Mechanism and design of** **allosteric activators of SIRT1**

Fei Liu^1,#^, Ning-Ning Pang^1,#^, Rui-Ming Xu^2^ and Na Yang^1,*^

1. State Key Laboratory of Medicinal Chemical Biology, College of Pharmacy and Key Laboratory of Medical Data Analysis and Statistical Research of Tianjin, Nankai University, 300353 Tianjin, China

2. National Laboratory of Biomacromolecules, CAS Center for Excellence in Biomacromolecules, Institute of Biophysics, Chinese Academy of Sciences, 100101 Beijing, China

#These authors contributed equally to this work.

*To whom correspondence should be addressed: [yangnanku@nankai.edu.cn](mailto:yangnanku@nankai.edu.cn)

**1 Methods**

**1.1 Simulation models**

Five structures were used in our simulations. The crystal structures of the SIRT1-Cp53-ac1 complex (PDBID: 4ZZJ) and the SIRT1-FDL-resveratrol complex (PDB ID: 5BTR) were the main reference structures (Cao et al., 2015; Dai et al., 2015). The former complex has an open NTD conformation, SIRT1 NTD is about 4.3 nm (centroid distance) from the SIRT1 CD, and ac1 interacts only with NTD (Fig. S13). The latter complex has a closed NTD conformation, resveratrol and NTD synergistically increase the binding of FDL to SIRT1, and there are very few direct contacts between NTD and CD, except for a hydrogen bond between E230 and R446 (Fig. S13). Mini-SIRT1 structures (containing minimal structural elements) were constructed, with a mini linker between the CD and the essential for SIRT1 activity (ESA) domain. The mini-SIRT1 can reveal the salient features of SIRT1 activation by the STACs and serve as an active and activatable surrogate for full-length SIRT1 (Cao et al., 2015; Dai et al., 2015). Structures that simulated the binding conformation of SIRT1 and three p53 peptides (N5p, N12p, and FDL) were constructed without STACs (Figs. S19, S20). SIRT1 was constructed (PDB ID: 4ZZJ) with the open NTD conformation to ensure that the simulation began with NTD not regulating CD. The SIRT1-p53 interactions were constructed according to the PDB structures 5BTR, 2H4J, and 1MA3 (Fig. S19) (Avalos et al., 2002; Hoff et al., 2006; Dai et al., 2015) and energy minimization was performed. Regarding the N5p and FDL peptides, the binding sites mainly involved SIRT1 N417 plus p53 K3, and SIRT1 R446 plus p53 L5 (or AMC), besides the acetylated lysine in the catalytic pocket (Fig. S20). Regarding the N12p peptide, the binding sites mainly involved SIRT1 G415, L416, and N417 plus p53 K6, and SIRT1 K444 and R446 plus p53 L8 and F10. These binding sites are consistent with the open NTD conformation of SIRT1 (PDB ID: 4ZZJ) and the structures of the homologous protein Sir2 (PDB ID: 2H4J and 1MA3), which lack NTD (Fig. S19). To achieve robust sampling regarding the SIRT1-p53 interactions and the related conformational changes, the REMD method was used (Zhou, 2007).

**1.2 Simulation Protocol**

For all models, the Amber99SB force field was used for the SIRT1 protein (Yu et al., 2018). The GAFF force field was used for the organic small molecules with AmberTools (Case et al., 2005). All simulations were performed with GROMACS 5.0.1 (Hess et al., 2008). A periodic cubic box with a margin of 1 nm was used, and the system was solvated using the TIP3P water model. Bonds involving hydrogens were constrained using the LINCS algorithm. A time-step of 2 fs was used. After energy minimization, a 1-ns NVT simulation and a 1-ns NPT simulation were performed on each system. A total of 6.1 μs of simulations was performed. For the non-REMD simulation systems, 200- or 70-ns dynamic simulations were performed at a temperature of 300 K. In the SMD simulations, to monitor the dynamics of the SIRT1-FDL-resveratrol complex (involving the opening of the closed NTD conformation in the presence of resveratrol) across a finite simulation time scale, pulling force simulations were used (Lu et al., 1998). In these SMD simulations, the umbrella force potentials were exerted on NTD with fast, moderate, and slow pulling rates of 0.015, 0.0081, and 0.00405 nm*ns^-1^, respectively. For the REMD simulations, 14 replicas were performed for each system at temperatures from 294.8 to 305.0 K (Fig. S21). The total simulation time for each system was 1.4 μs. For the REMD simulations, the exchange frequency between neighbor replicas was every 1000 steps and the exchange probabilities were around 0.2, guaranteeing REMD sampling efficiency (Fig. S21). Finally, all the trajectories were collected, and the weighted histogram analysis method (WHAM) was used to obtain the thermodynamic results (Chodera et al., 2007).

**1.3 HMD method**

The compounds in a virtual compound library (Specs) were used as the ligands for docking. All molecular dockings were performed using AutoDock 4.2 (Goodsell et al., 1996). For docking in modified close conformation, the docking grid was centered on the geometric center of the leucine (Fig. S13C). The diameter of docking grid was set as about 1.8 nm. For docking in open conformation, the grid box was centered on the binding geometric center of ac1 and NTD. The docking gird size was set as 60*60*60 grids with a grid spacing of 0.375. The box was large enough to cover the potential binding surface of the STAC. The HMD processes were controlled by in-house scripts. Each docking ligand was considered conformationally flexible, with its torsional bonds defined by AutoDock 4.2 according to the chemical features. The decoy with the lowest binding free energy was saved as the final ligand docking pattern with the receptor.

**1.4 Protein expression and purification**

Human SIRT1-143CS (a truncated variant with four cysteine-to-serine mutations) gene was cloned into a pET28a-smt3 vector and expressed in *E. coli* BL21(DE3) cells as described previously (Cao et al., 2015). The cells were harvested by centrifugation and resuspended in ice-cold lysis buffer containing 20 mM Tris-HCl (pH 8.0), 500 mM NaCl, 5% glycerol and 1 mM phenylmethylsulfonyl fluoride (PMSF). The cells were lysed by sonication and then underwent further centrifugation. The supernatant was loaded into a Ni-NTA affinity column and eluted via a 0*–*100 mM imidazole gradient in 20 mM Tris-HCl (pH 8.0), 200 mM NaCl, and 5% glycerol. The complex eluted from the affinity column was digested using SUMO protease for 2 h at 4℃, and then the sample was diluted and reloaded into the Ni-NTA column to remove the His-SUMO tags. The tag-free proteins were concentrated and then further purified using a Superdex HiLoad 75 16/60 size exclusion column (GE Healthcare) in a buffer containing 20 mM HEPES (pH 7.5), 150 mM NaCl, 10% glycerol, and 0.1% *β*-mercaptoethanol. The purified SIRT1-143CS protein was collected and stored at -80℃.

**1.5 *In vitro* ECD assays**

The deacetylase activity of SIRT1 was measured using an ECD microplate assay (Garrity et al., 2007; Smith et al., 2009; Cao et al., 2015). In the assays, the deacetylation reaction product nicotinamide was converted to nicotinic acid and ammonia by nicotinamidase (PncA). Next, glutamate dehydrogenase converted the ammonia, α-ketoglutarate, and NADH to glutamate and NAD^+^. The oxidation of NADH was measured spectrophotometrically at 340 nm to reflect the molality of the deacetylation reaction product nicotinamide. The molality, c, was determined by c=kd/A, where A is the absorbance at 340 nm, k is the molar absorption coefficient, and d is the optical path length (Cao et al., 2015). Finally, the reaction rate was calculated as the molality divided by time. The SIRT1 deacetylase activity in the presence of various STAC concentrations was measured, while the remaining conditions were kept constant. The assays involved adding 400 nM SIRT1-143CS, 100 μM substrate peptides, 0.2 mM NADH, 3.3 mM α-ketoglutarate, 1 μM nicotinamidase (PncA), 3 U bovine liver glutamate dehydrogenase (Sigma), 1 mM dithiothreitol (DTT), and 2% dimethyl sulfoxide (DMSO) to PBS (pH 7.4) at 37℃ for 10-5 min until the readings at 340 nm reached equilibrium, and the reactions were then initiated by adding 2 mM NAD^+^. The assays were performed in triplicate. And compounds were bought from Topscience company (Shanghai, China).

**1.6 *In cell* deacetylase assays of SIRT1 with STACs**

Deacetylase assays of SIRT1 were performed at HEK293T, HeLa and SIRT1 knockout HeLa cells (ABclonal Biotechnology Co.,Ltd ) as previous reported (Hassig et al., 1997; Vaquero et al., 2004). In brief, a luciferase reporter plasmid pGL6 (Beyotime, China) with a mini TA promoter was constructed by adding five consensus binding sites of *S. cerevisiae* Gal4 DNA binding domain (a.a.1-147), acting as a transcriptional activator (Fig. S22). The SIRT1-143-CS was fused to the Gal4 DNA binding domain and cloned to pcDNA5. When expressed, the Gal4-SIRT1 fusion protein bound to the 5x Gal4 binding sites, recruiting SIRT1 to the TA promoter of the luciferase reporter. If STACs activate the deacetylation of SIRT1, the expression of luciferase gene will be repressed (Fig. S22). The expression level of luciferase was detected by luminescence reaction. In detail, 50 μl supernatant of cell extraction was incubated with 10 μM ATP and 10 mM MgCl_2_ in 96-well white plates, luminescence were detected at 360-700 nm in a microplate reader (TECAN, Austria), the reaction was initiated by adding 10 μM luciferin. The pCMV-β-galactosidase (pCMV-β-gal) was expressed as transfection control gene, and the level of luciferase activity was normalized to the level of β-galactosidase activity (Herbomel et al., 1984).

**1.7 References**

Avalos, J.L., Celic, I., Muhammad, S., Cosgrove, M.S., Boeke, J.D., and Wolberger, C. (2002). Structure of a Sir2 enzyme bound to an acetylated p53 peptide. Mol Cell 10, 523-535.

Cao, D., Wang, M., Qiu, X., Liu, D., Jiang, H., Yang, N., and Xu, R.M. (2015). Structural basis for allosteric, substrate-dependent stimulation of SIRT1 activity by resveratrol. Genes Dev 29, 1316-1325.

Case, D.A., Cheatham, T.E., 3rd, Darden, T., Gohlke, H., Luo, R., Merz, K.M., Jr., Onufriev, A., Simmerling, C., Wang, B., and Woods, R.J. (2005). The Amber biomolecular simulation programs. J Comput Chem 26, 1668-1688.

Chodera, J.D., Swope, W.C., Pitera, J.W., Seok, C., and Dill, K.A. (2007). Use of the Weighted Histogram Analysis Method for the Analysis of Simulated and Parallel Tempering Simulations. J Chem Theory Comput 3, 26-41.

Dai, H., Case, A.W., Riera, T.V., Considine, T., Lee, J.E., Hamuro, Y., Zhao, H., Jiang, Y., Sweitzer, S.M., Pietrak, B.*, et al.* (2015). Crystallographic structure of a small molecule SIRT1 activator-enzyme complex. Nat Commun 6, 7645.

Garrity, J., Gardner, J.G., Hawse, W., Wolberger, C., and Escalante-Semerena, J.C. (2007). N-lysine propionylation controls the activity of propionyl-CoA synthetase. J Biol Chem 282, 30239-30245.

Goodsell, D.S., Morris, G.M., and Olson, A.J. (1996). Automated docking of flexible ligands: applications of AutoDock. J Mol Recognit 9, 1-5.

Hassig, C.A., Fleischer, T.C., Billin, A.N., Schreiber, S.L., and Ayer, D.E. (1997). Histone deacetylase activity is required for full transcriptional repression by mSin3A. Cell 89, 341-347.

Herbomel, P., Bourachot, B., and Yaniv, M. (1984). Two distinct enhancers with different cell specificities coexist in the regulatory region of polyoma. Cell 39, 653-662.

Hess, B., Kutzner, C., van der Spoel, D., and Lindahl, E. (2008). GROMACS 4: Algorithms for Highly Efficient, Load-Balanced, and Scalable Molecular Simulation. J Chem Theory Comput 4, 435-447.

Hoff, K.G., Avalos, J.L., Sens, K., and Wolberger, C. (2006). Insights into the sirtuin mechanism from ternary complexes containing NAD+ and acetylated peptide. Structure 14, 1231-1240.

Lu, H., Isralewitz, B., Krammer, A., Vogel, V., and Schulten, K. (1998). Unfolding of titin immunoglobulin domains by steered molecular dynamics simulation. Biophys J 75, 662-671.

Smith, B.C., Hallows, W.C., and Denu, J.M. (2009). A continuous microplate assay for sirtuins and nicotinamide-producing enzymes. Anal Biochem 394, 101-109.

Vaquero, A., Scher, M., Lee, D., Erdjument-Bromage, H., Tempst, P., and Reinberg, D. (2004). Human SirT1 interacts with histone H1 and promotes formation of facultative heterochromatin. Mol Cell 16, 93-105.

Yu, I., Feig, M., and Sugita, Y. (2018). High-Performance Data Analysis on the Big Trajectory Data of Cellular Scale All-atom Molecular Dynamics Simulations. J Phys Conf Ser 1036.

Zhou, R. (2007). Replica exchange molecular dynamics method for protein folding simulation. Methods Mol Biol 350, 205-223.

| FDL (p53) | Arg-His-Lys-Lys(Ac)-AMC |
| --- | --- |
| FDL (p53) in SMD | Ac-Arg-His-Lys-Lys(Ac)-AMC |
| Cp53 (p53) | Arg-His-Lys-Lys(Ac)-Leu-NLE-Phe |
| N5p (p53) | Arg-His-Lys-Lys(Ac)-Leu |
| N12p (p53) | Ser-Thr-Ser-Arg-His-Lys-Lys(Ac)-Leu-Met-Phe-Lys-Thr |
| NAp (p53) | Arg-His-Lys-Lys(Ac) |
| PGC1α | Pro-Ala-Ser-Thr-Lys-Ser-Lys(Ac)-Tyr-Asp-Ser-Leu-Asp-Phe |
| FOXO3a | Ser-Pro-Ser-Gln-Leu-Ser-Lys(Ac)-Trp-Pro-Gly-Ser-Pro |

Table S1. Sequences of the substrate peptides studied in the present study. Lys(Ac) is acetylated lysine, and NLE is a modified norleucine with a longer side chain that occurred during crystallization (but remained hydrophobic with an aliphatic side chain).

| **Name** | **Autodock_energy (kcal/mol)** |
| --- | --- |
| STAC-n12p-1 | -10.60 |
| STAC-n12p-2 | -10.09 |
| STAC-n12p-3 | -9.53 |
| STAC-n12p-4 | -9.27 |
| STAC-n12p-5 | -10.12 |
| STAC-n12p-6 | -9.44 |
| STAC-n12p-7 | -9.40 |
| STAC-n12p-8 | -12.02 |
| STAC-n12p-9 | -10.89 |
| STAC-n12p-10 | -11.11 |
| STAC-n12p-11 | -11.15 |
| STAC-n12p-12 | -10.99 |
| STAC-n12p-13 | -9.63 |
| STAC-n12p-14 | -9.94 |
| STAC-n12p-15 | -8.89 |
| STAC-n12p-16 | -10.18 |
| STAC-n12p-17 | -10.31 |
| STAC-n12p-18 | -10.24 |
| STAC-n12p-19 | -10.57 |
| STAC-n12p-20 | -10.21 |

Table S2. The binding free energy (Autodock_energy) of the selected 20 compounds.


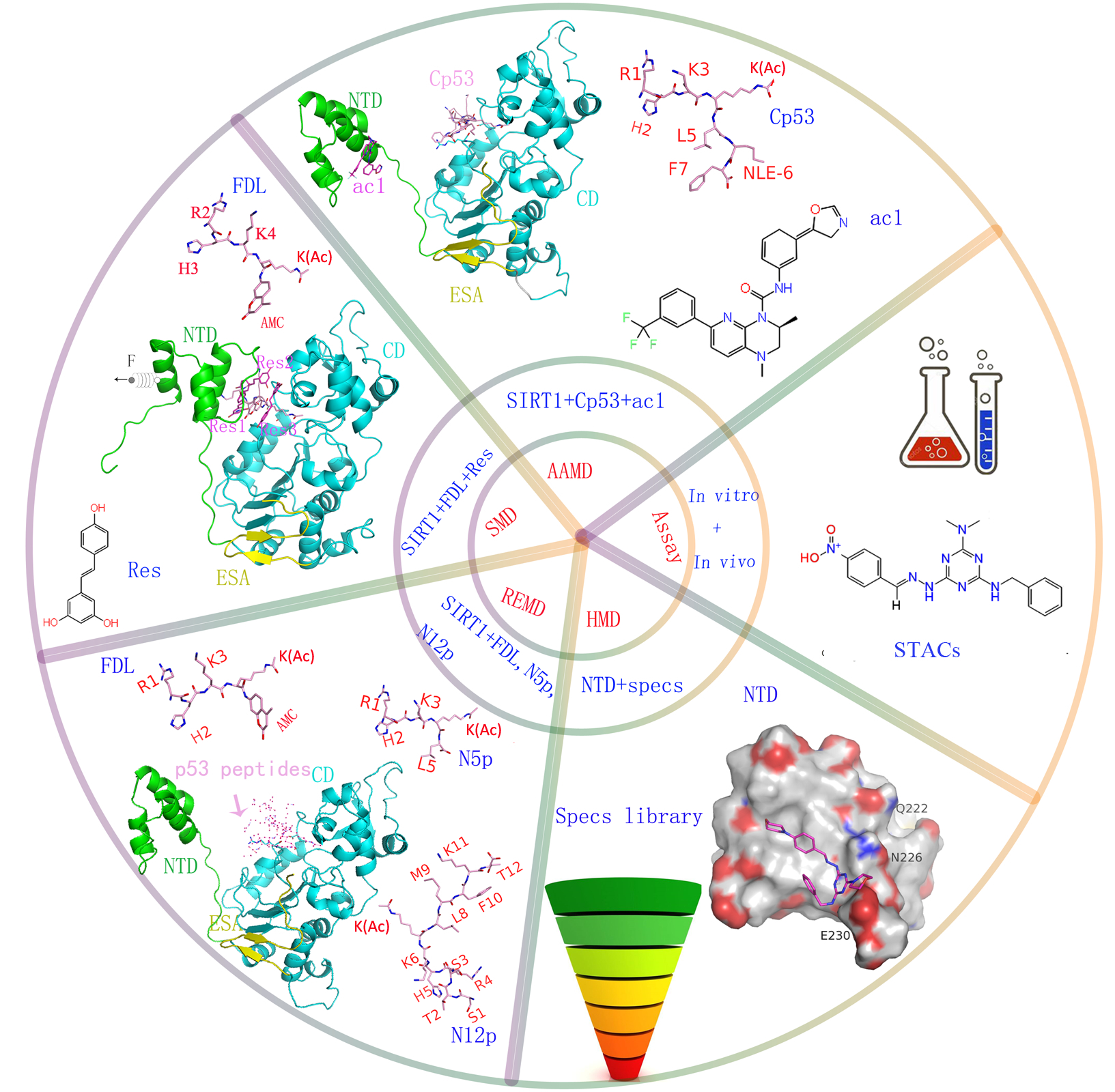


Figure S1. Overview of methods used in this study. AAMD: all-atom molecular dynamics simulations to explore the allosteric activation of the SIRT1-Cp53-ac1 complexes. SMD: steered molecular dynamics simulations to explore the allosteric activation of the SIRT1-FDL-resveratrol complex. REMD: replica exchange molecular dynamics simulation to study the SIRT1-p53 (FDL, N5p and N12p) interactions without STACs. HMD: high-throughput molecular docking (HMD) to search the virtual compound library. Specs, to find lead compounds that can bind to SIRT1 NTD. Assay: deacetylation assay of SIRT1 with various substrates and STACs *in vitro* and *in cell*. The colors indicate the followings: green: SIRT1 NTD; cyan: SIRT1 CD; yellow: SIRT1 ESA domain; magenta: resveratrols (Res1, Res2, and Res3) and ac1 (STACs); salmon: p53 peptides. The two-dimensional chemical formula of each STAC is shown.


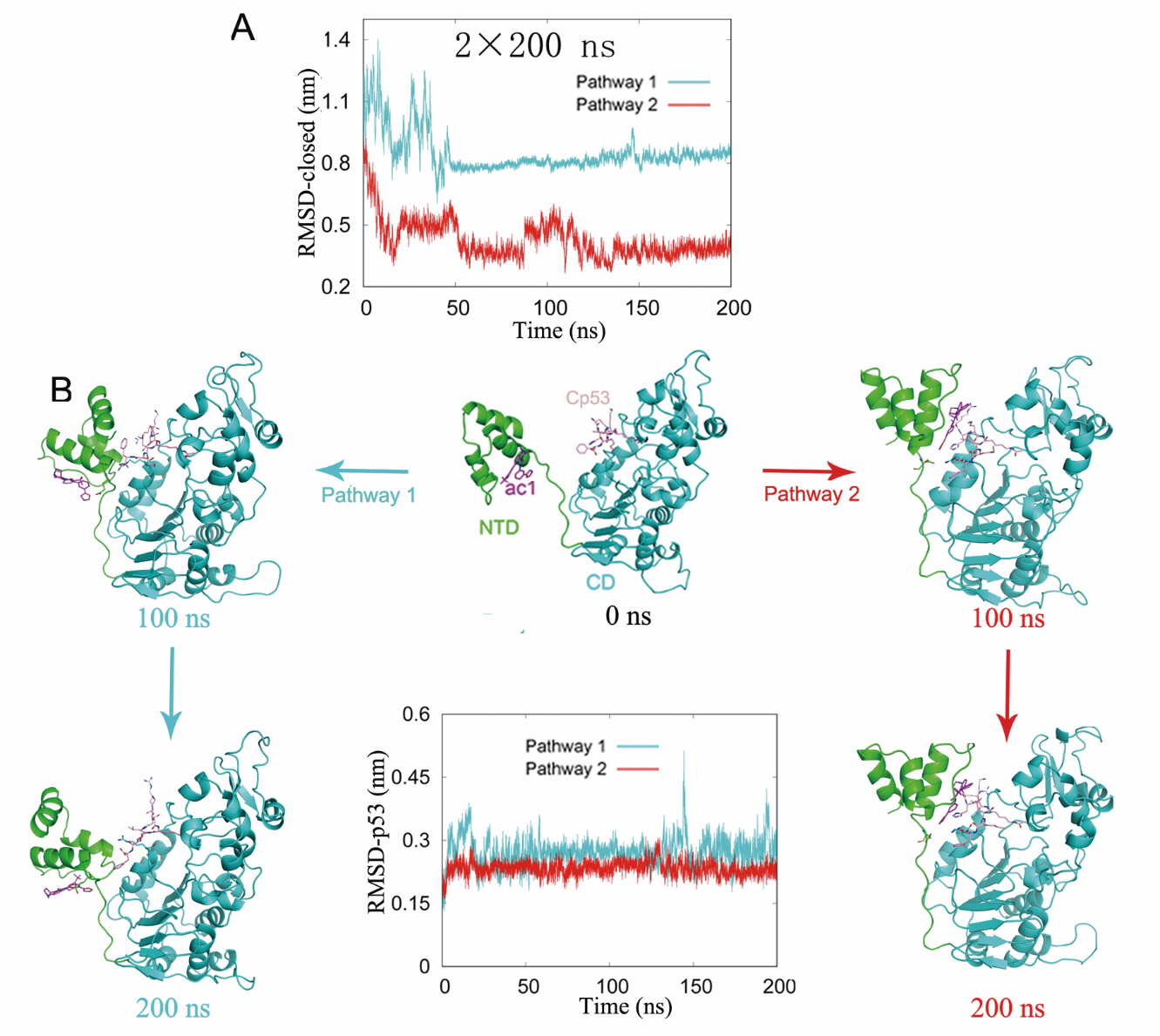


Figure S2. The results of AAMD. (A) Two independent 200-ns simulations. Cyan line: dynamic pathway 1; red line: dynamic pathway 2. RMSD-closed is the SIRT1 root-mean-square deviation (RMSD) values relative to the closed SIRT1 conformation (PDB ID: 5BTR). (B) Structures from two dynamic pathways after 100-ns and 200-ns of simulations are shown respectively. RMSD-p53 is the RMSD values of p53 peptide relative to the initial location of Cp53 in the open conformation. Salmon: p53 peptide; magenta: ac1; green: SIRT1 NTD; cyan: SIRT1 CD; cyan arrows: dynamic pathway 1; red arrows: dynamic pathway 2.


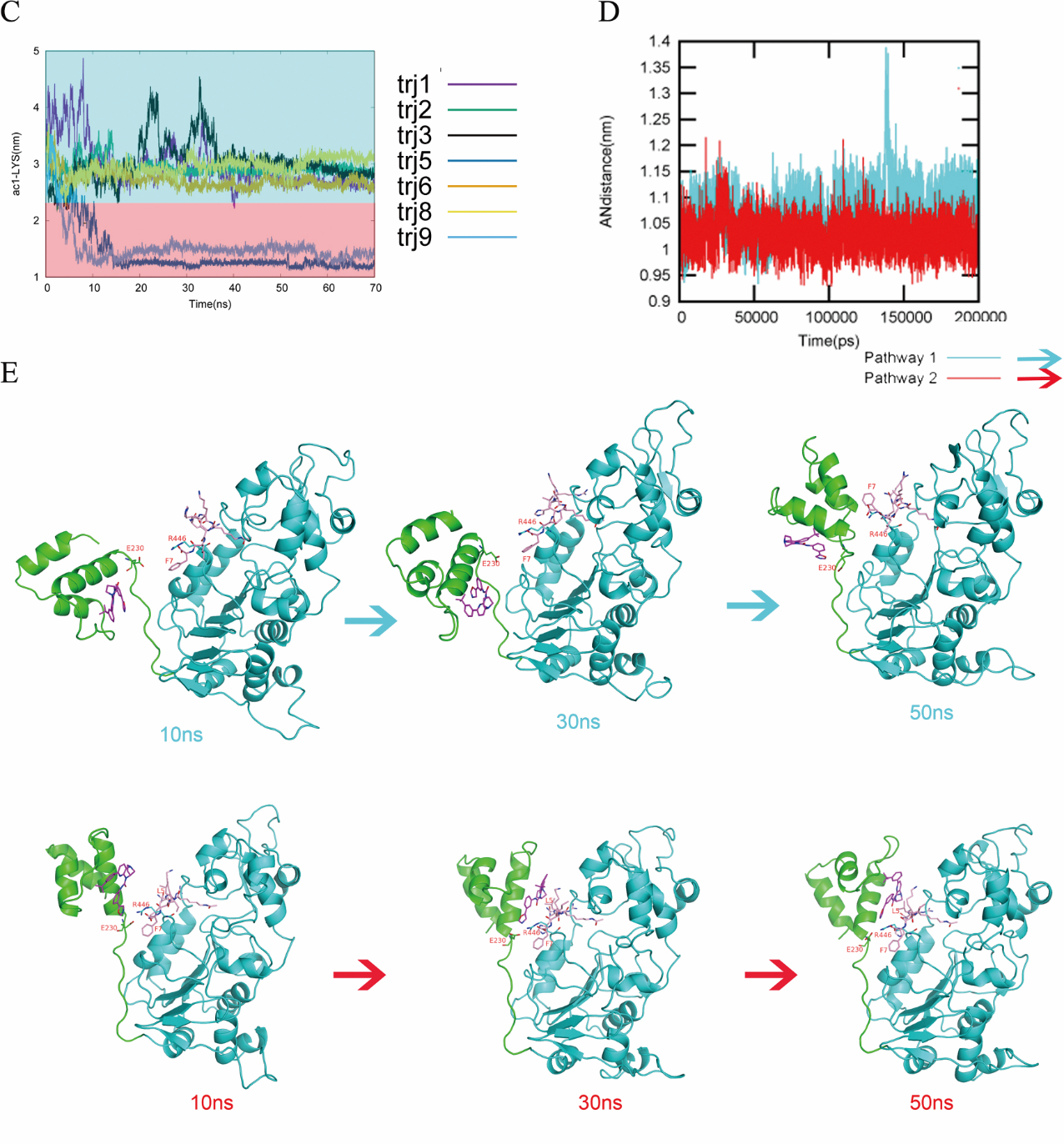


Figure S2. The results of AAMD. (C) The seven 70-ns trajectories of the ac1-LYS of SIRT1-p53-ac1 system are shown. The ac1-LYS represent the centroid distance between STACs ac1 and acetylated lysine. (D) The trajectories of the ANdistance and structures of SIRT1-p53-ac1 system are shown. The ANditance stands for the distance between the NTD domain of SIRT1 and the activator ac1. The cyan and red line trajectories of the ANdistance stand for the dynamic pathway 1 and pathway 2. (E) Representative structures extracted from dynamic pathway (10 ns - 50 ns). The cyan and red arrow stand for the dynamic pathway 1 and pathway 2.


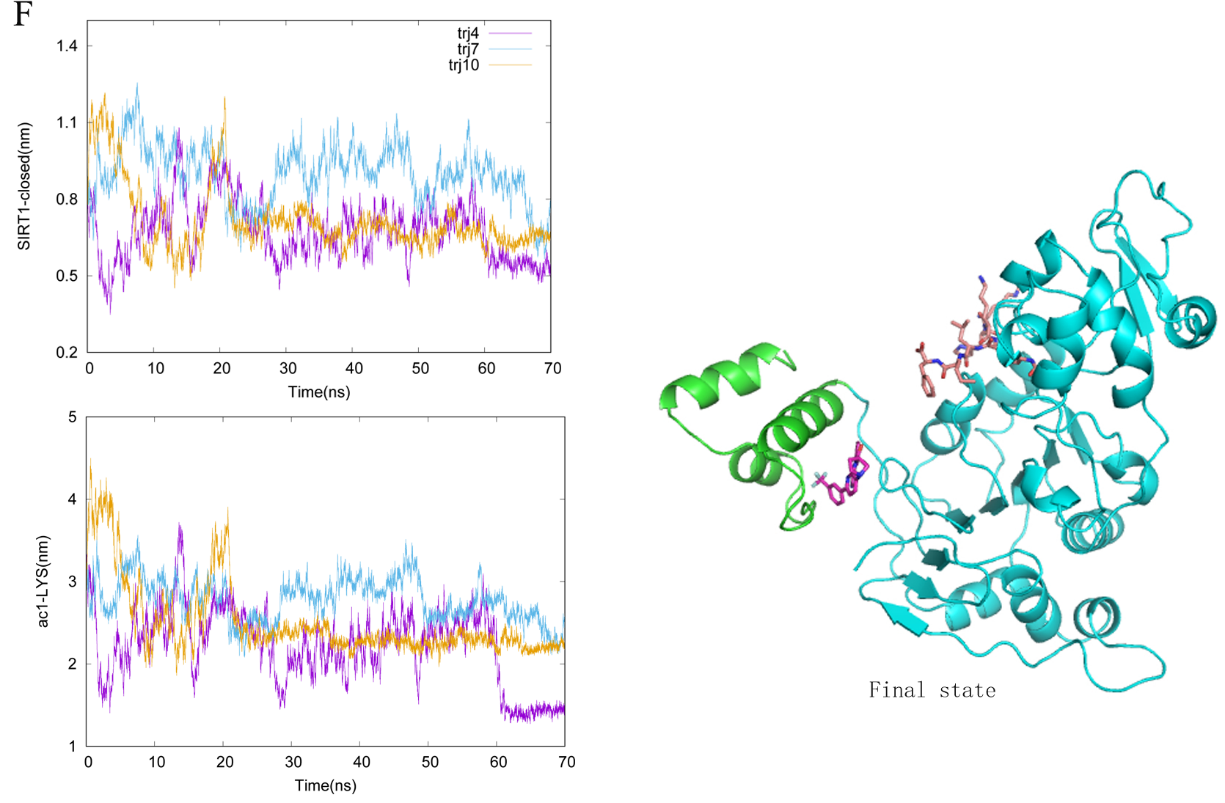


Figure S2. The results of AAMD. (F) The RMSD-closed represent the Root-Mean-Square Deviation values of the SIRT1 relative to SIRT1 closed conformation in structure 5BTR. The ac1-LYS represent the centroid distance between STACs ac1 and acetylated lysine. The final stable state was shown in the cartoon model.


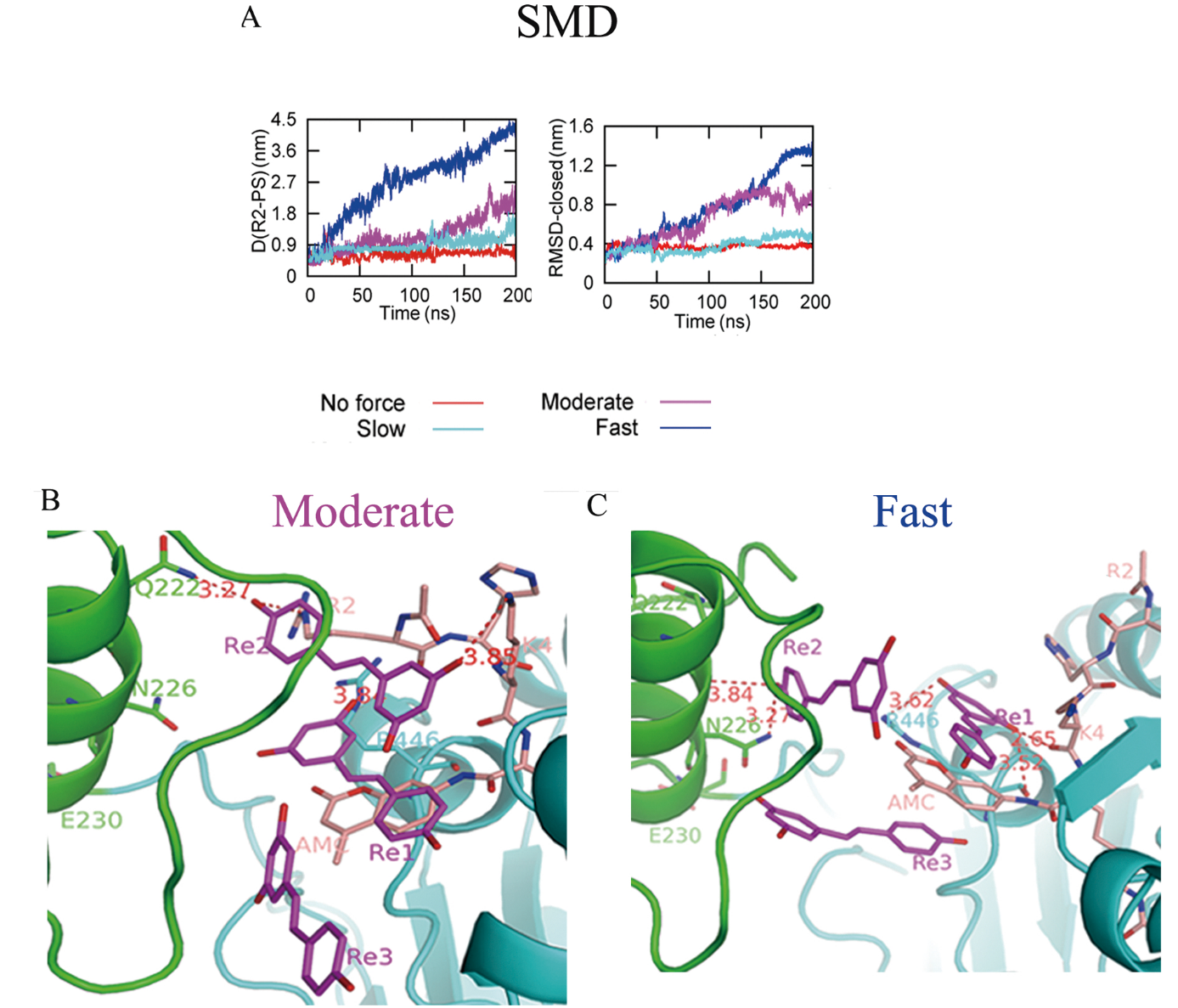


Figure S3. The results of SMD. (A) Dynamic trajectory of the SIRT1-FDL-resveratrol system with AAMD (red: no force) and SMD (cyan: slow pulling rate, magenta: moderate pulling rate, blue: fast pulling rate). RMSD-closed is the SIRT1 RMSD values relative to the closed conformation (PDB structure 5BTR). D(R2-PS) is the centroid distances between R2 of FDL and SIRT1 residues Q222 and N226. (B-C) Structures extracted from the moderate and fast pulling SMD simulations, respectively. Salmon: p53 peptide; magenta: resveratrols; green: SIRT1 NTD; cyan: SIRT1 CD.


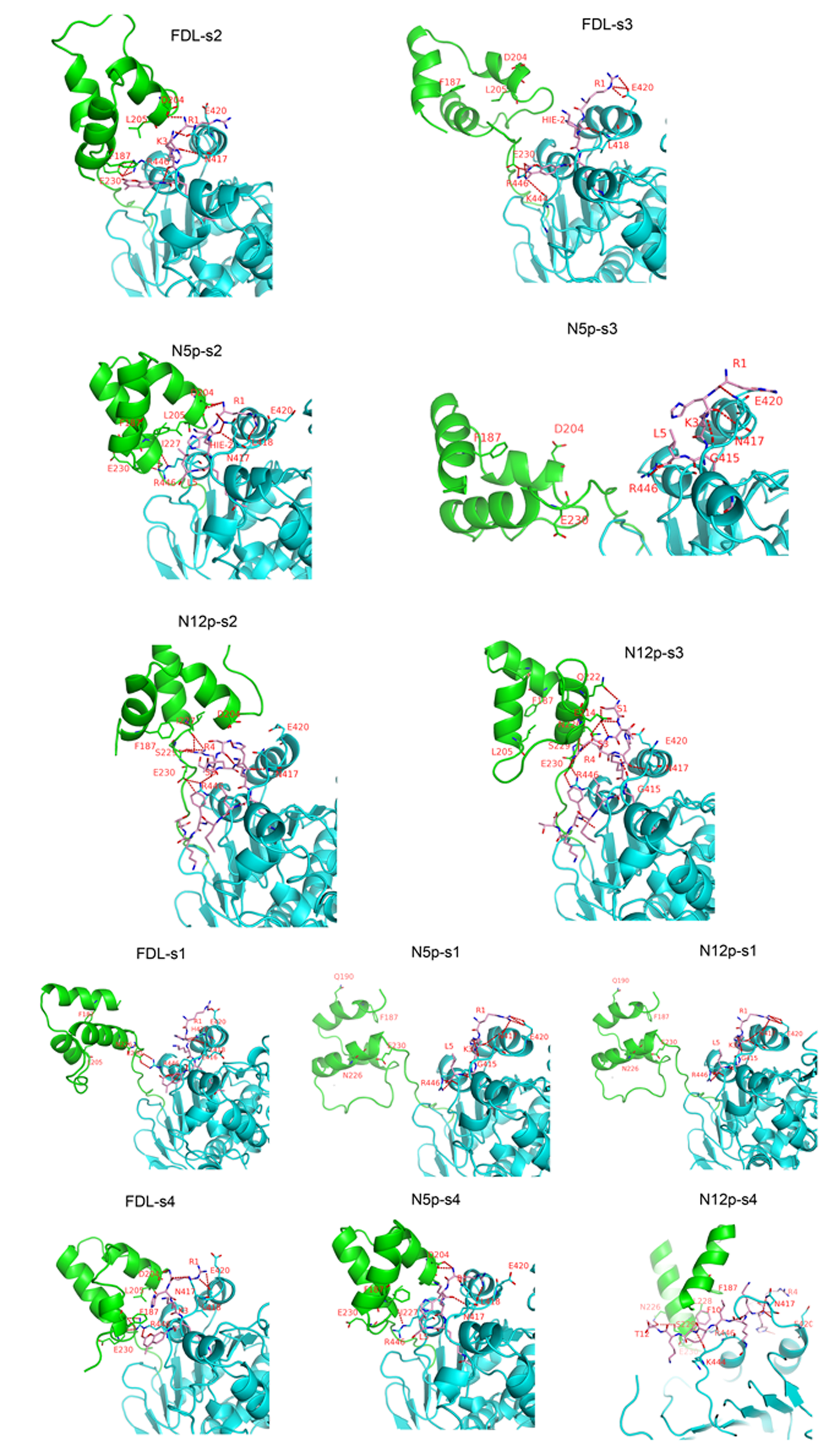


Figure S4. Structures extracted from different thermodynamic states. The NTD and CD domain of SIRT1 are colored green and cyan, respectively. The p53 is colored salmon. Hydrogen bonds are shown by red dotted lines.


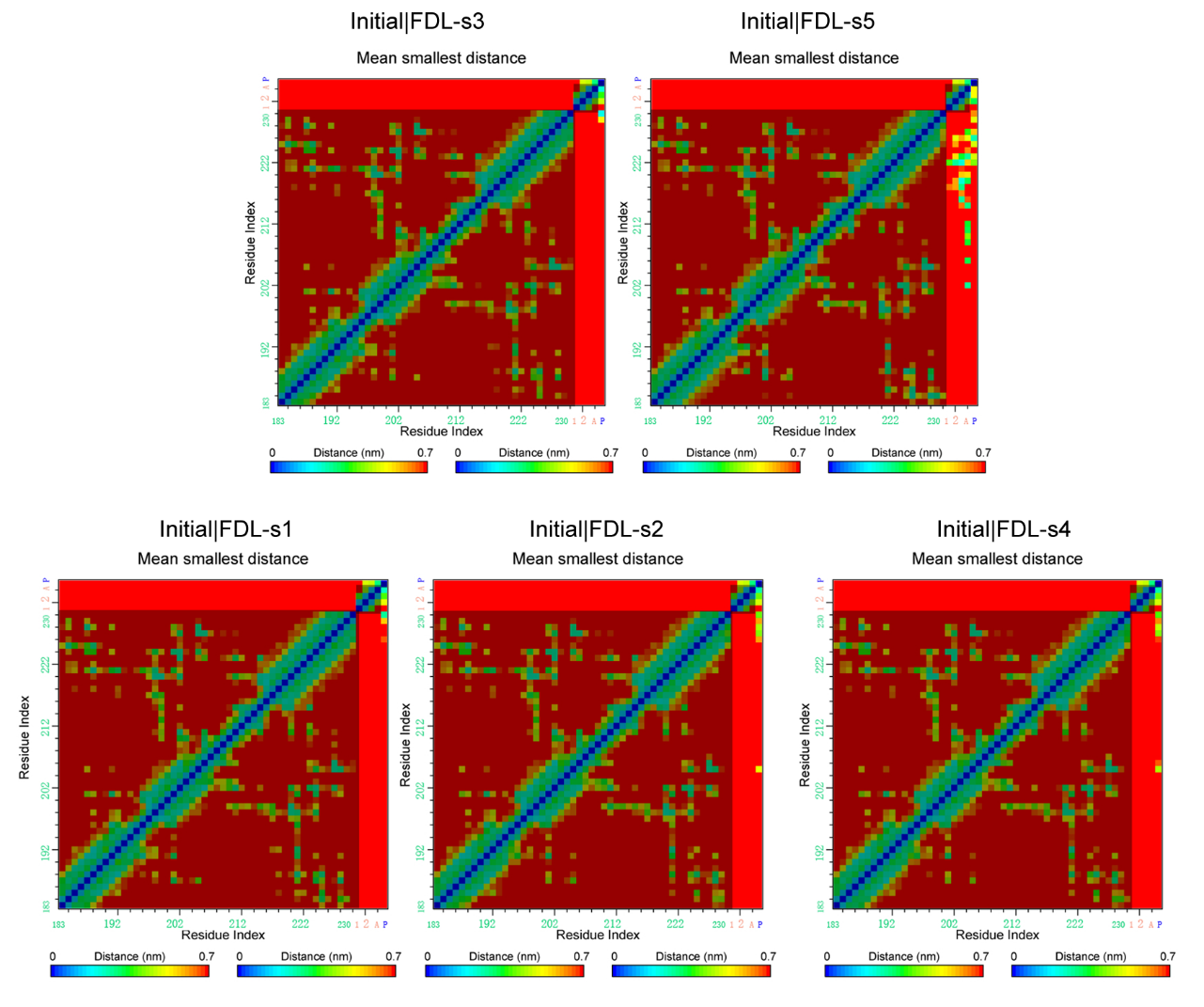


Figure S5. The contact map of thermodynamic states of FDL-s1, -s2, -s3, -s4 and s5. The contact between each two residues was calculated by the smallest distance between all atoms of each two residues and the cut-off was 0.7 nm. The contact map was constructed for each thermodynamic state by getting the mean smallest distance of all structures in the thermodynamic states. The residue index of NTD was colored green in axis. The residue index of p53 was colored by salmon and the residue index of R446(p) was colored by blue. The intra-domain contacts were marked by shadow.


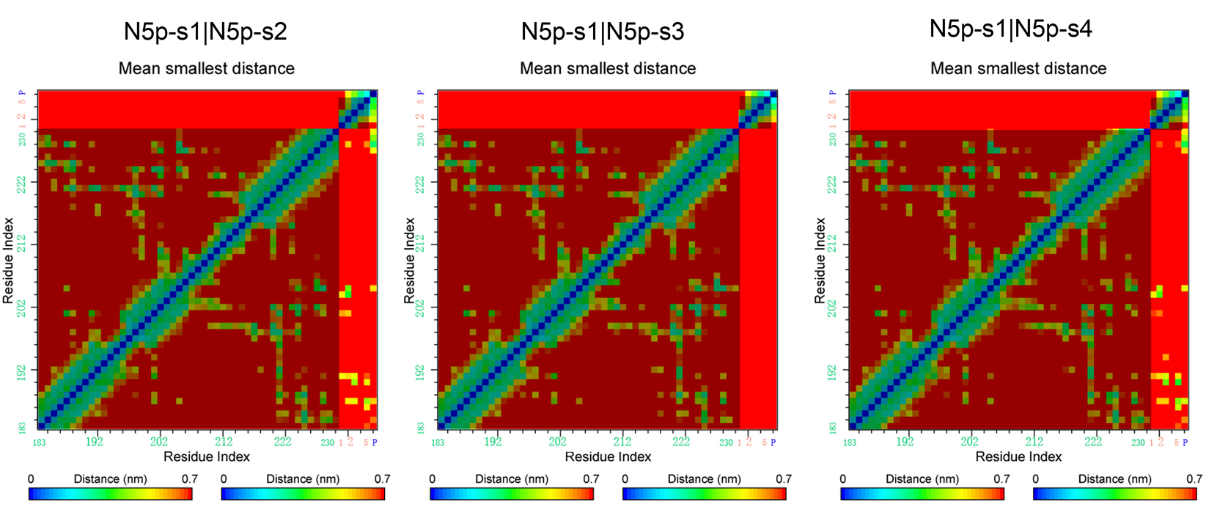


Figure S6. The contact map of thermodynamic states of N5p-s2, -s3 and -s4. The contact between each two residues was calculated by the smallest distance between all atoms of each two residues and the cut-off was 0.7 nm. The contact map was constructed for each thermodynamic state by getting the mean smallest distance of all structures in the thermodynamic states.


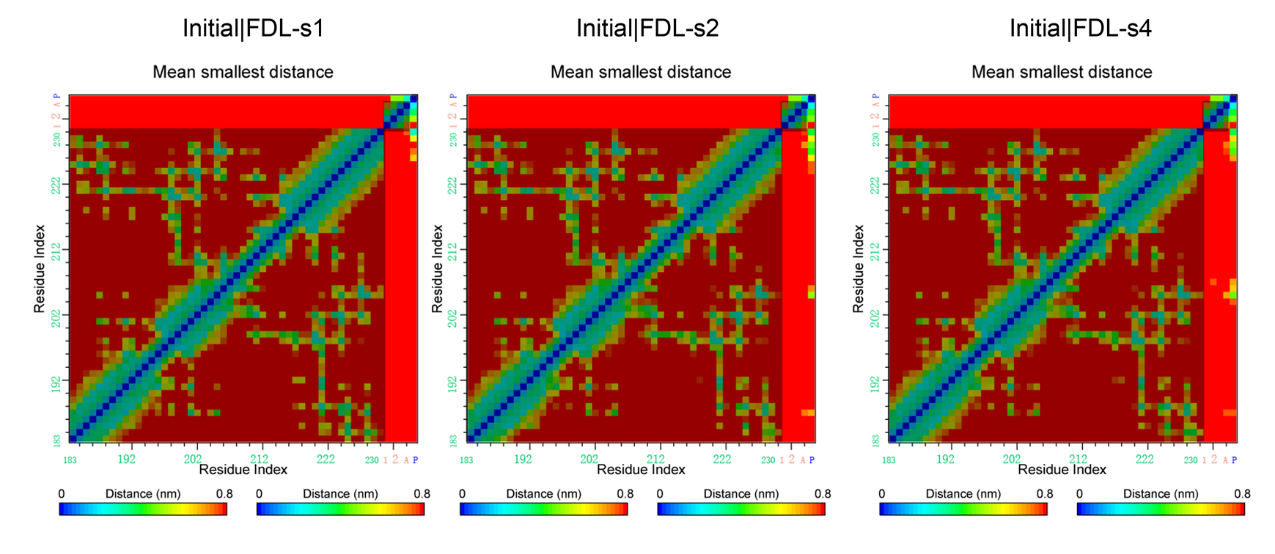


Figure S7. The contact map of thermodynamic states of FDL-s1, -s2 and -s4. The contact between each two residues was calculated by the smallest distance between all atoms of each two residues and the cut-off was 0.8 nm. The contact map was constructed for each thermodynamic state by getting the mean smallest distance of all structures in the thermodynamic states.
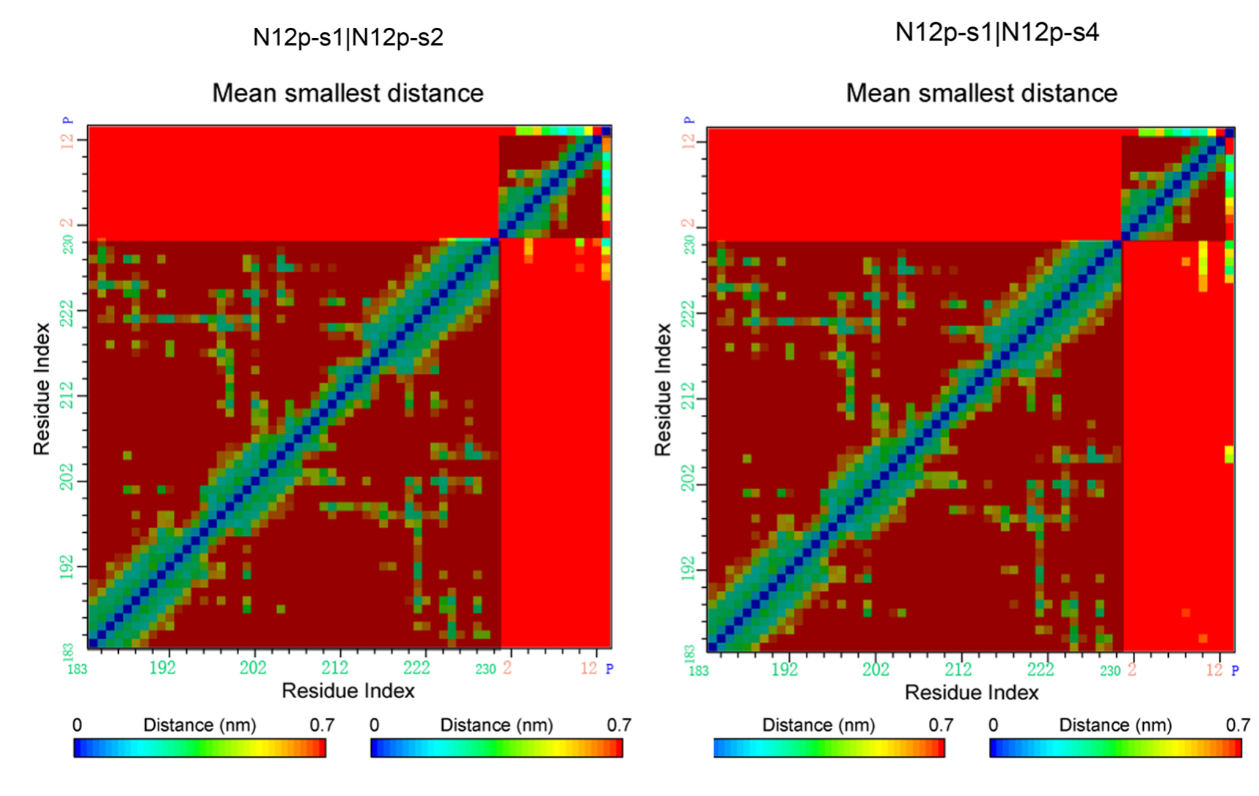


Figure S8. The contact map of thermodynamic states of N12p-s2 and -s4. The contact between each two residues was calculated by the smallest distance between all atoms of each two residues and the cut-off was 0.7 nm. The contact map was constructed for each thermodynamic state by getting the mean smallest distance of all structures in the thermodynamic states. The residue index of NTD was colored green in axis. The residue index of p53 was colored by salmon and the residue index of R446(p) was colored by blue. The intra-domain contacts were marked by shadow.


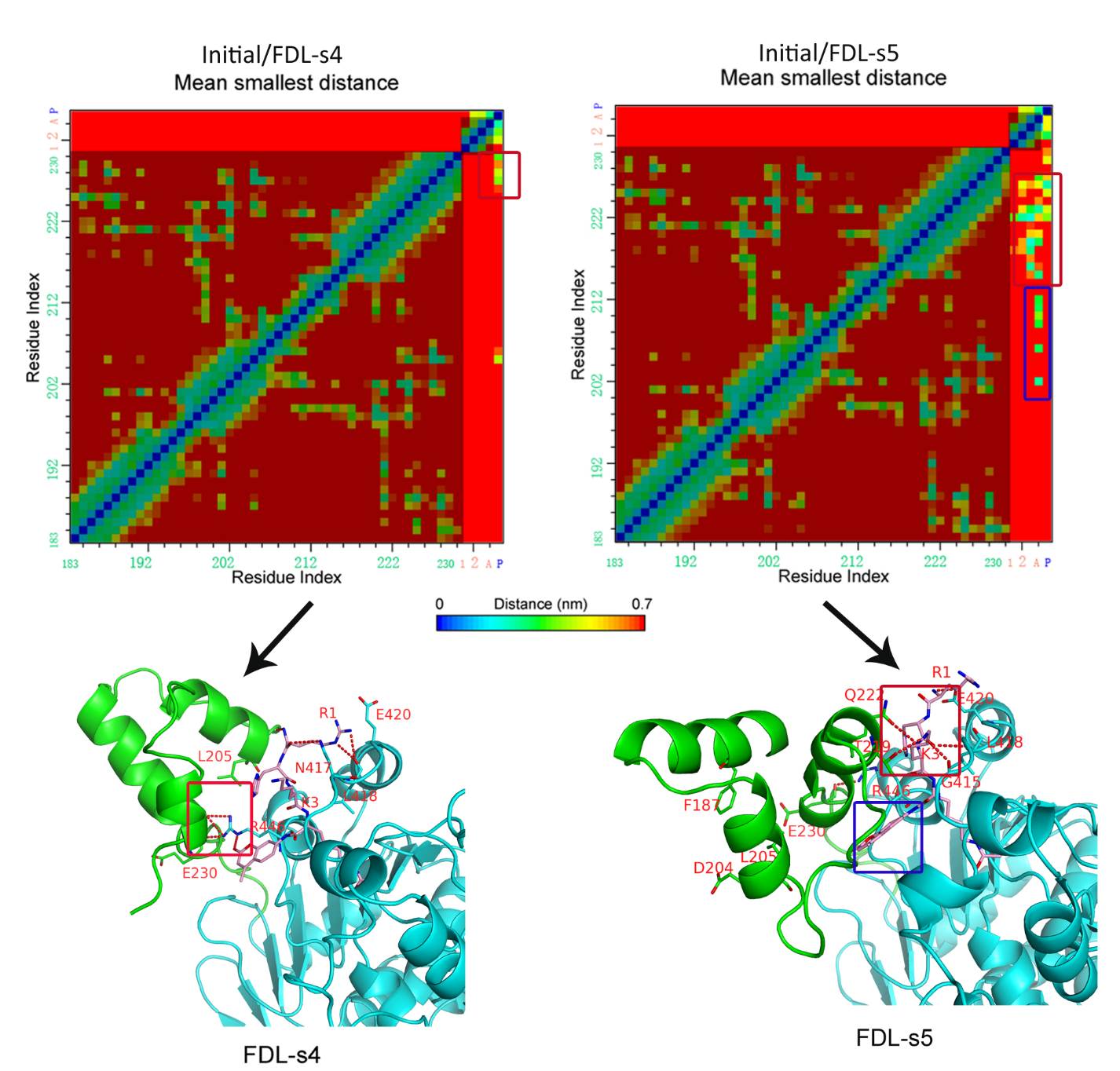


Figure S9. Contact maps of the thermodynamic states of FDL-s4 and FDL-s5 (Contact maps in the initial state of simulation are shown in the upper panel). Contacts between each pair of residues was calculated based on the smallest distance between all atoms of each pair of residues and the cut-off was 0.7 nm. Green, salmon, and blue on the axes indicate the residue index of NTD, p53, and R446(p), respectively. Shadowing indicates the inter-domain contacts. Structures from different thermodynamic states are shown at the bottom (SIRT1 is shown using a cartoon model, while the p53 peptides and residues in the binding sites are shown in stick models). Green: SIRT1 NTD; cyan: SIRT1 CD; salmon: p53 peptides; red dotted line: hydrogen bonds; blue boxes: NTD SBHS contacts; red boxes: NTD SBPS contacts. NTD: N-terminal domain; SBHS: STAC-binding hydrophobic site; SBPS: STAC-binding polar site; SIRT1: sirtuin 1; STAC: sirtuin-activating compound.


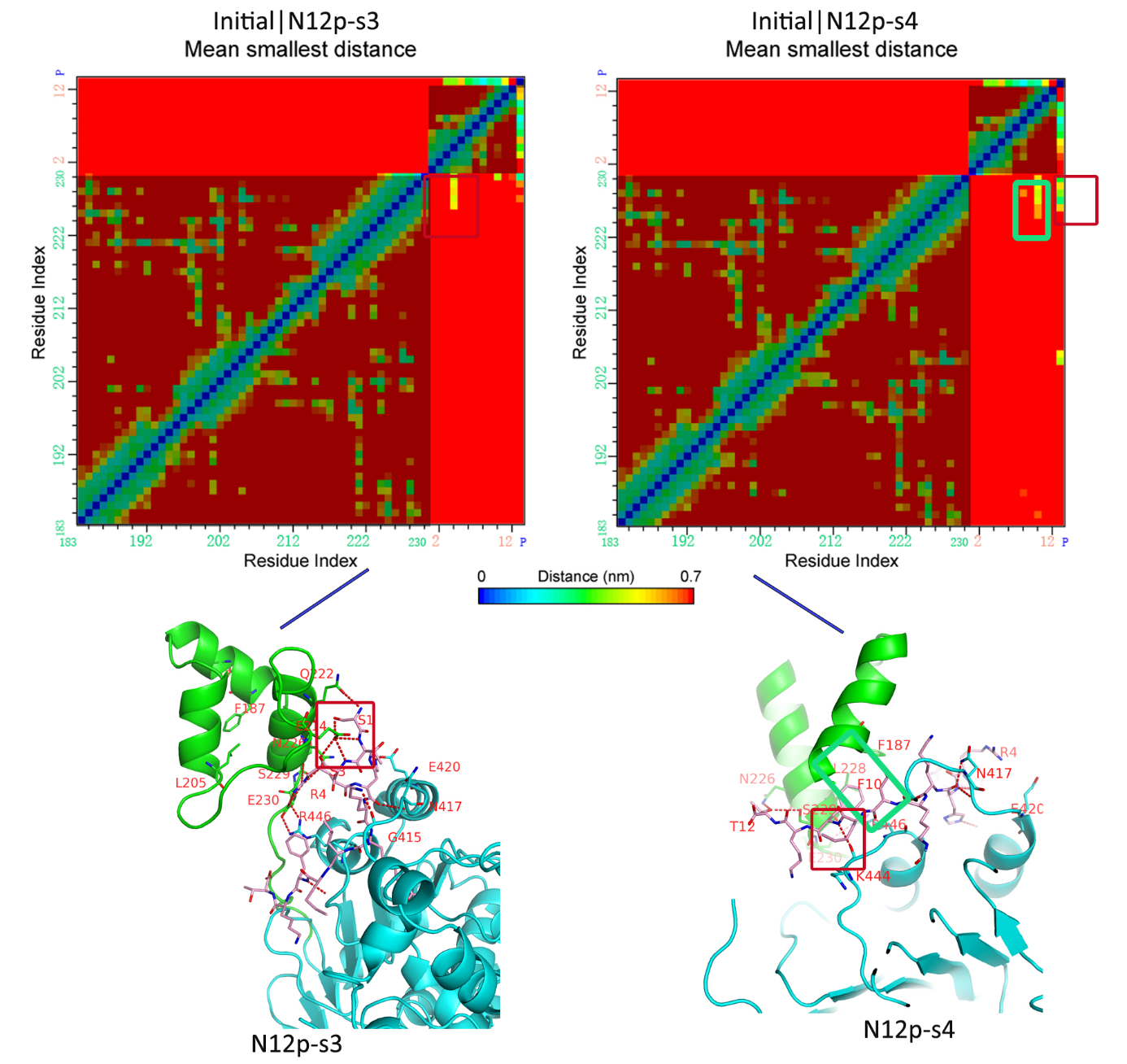
Figure S10. The contact maps of thermodynamic states of N12p-s3 and N12p-s4 state. The contacts between each two residues was calculated by the smallest distance between all atoms of each two residues and the cut-off was 0.7 nm. The residue index of NTD was colored green in axis. The residue index of p53 was colored by salmon and the residue index of R446(p) was colored by blue. The intra-domain contacts were marked by shadow. Structures extracted from different thermodynamic states are shown by cartoon model. The p53 peptides and residues in the binding sites are shown by stick model. The NTD and CD domain of SIRT1 are colored green and cyan, respectively. The p53 peptides is colored salmon. Hydrogen bonds are shown by red dotted lines. The contacts involved in NSHS and SBPS are marked by green and red boxes, respectively.


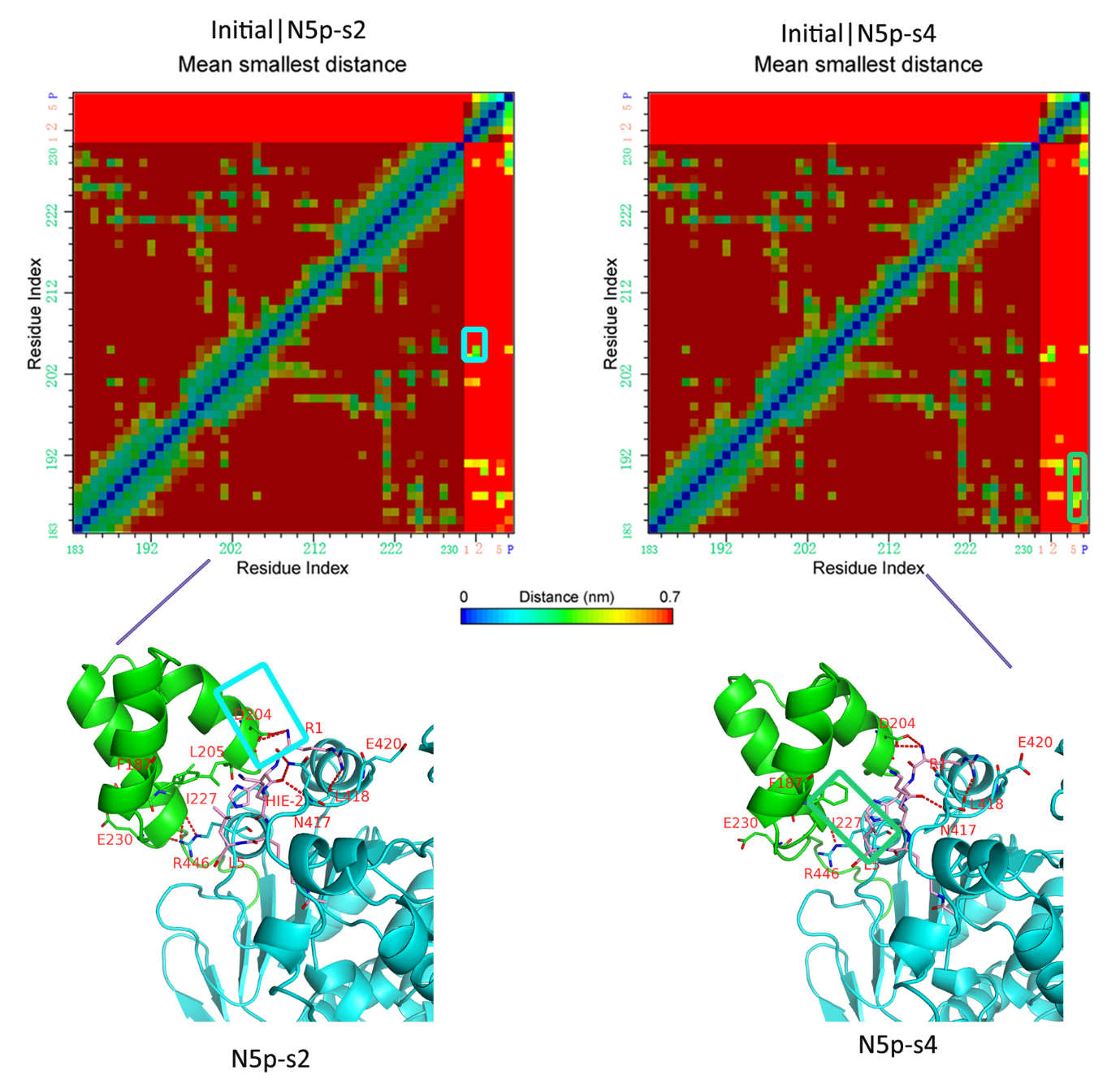


Figure S11. The contact maps of thermodynamic states of N5p-s2 and N5p-s4 state. The contacts between each two residues was calculated by the smallest distance between all atoms of each two residues and the cut-off was 0.7 nm. The residue index of NTD was colored green in axis. The residue index of p53 was colored by salmon and the residue index of R446(p) was colored by blue. The intra-domain contacts were marked by shadow. Structures extracted from different thermodynamic states are shown by cartoon model. The p53 peptides and the residues in the binding sites are shown by stick model. The NTD and CD domain of SIRT1 are colored green and cyan, respectively. The p53 peptides is colored salmon. Hydrogen bonds are shown by red dotted lines. The contacts involved in NSBS and NSPS are marked by green and cyan boxes, respectively.


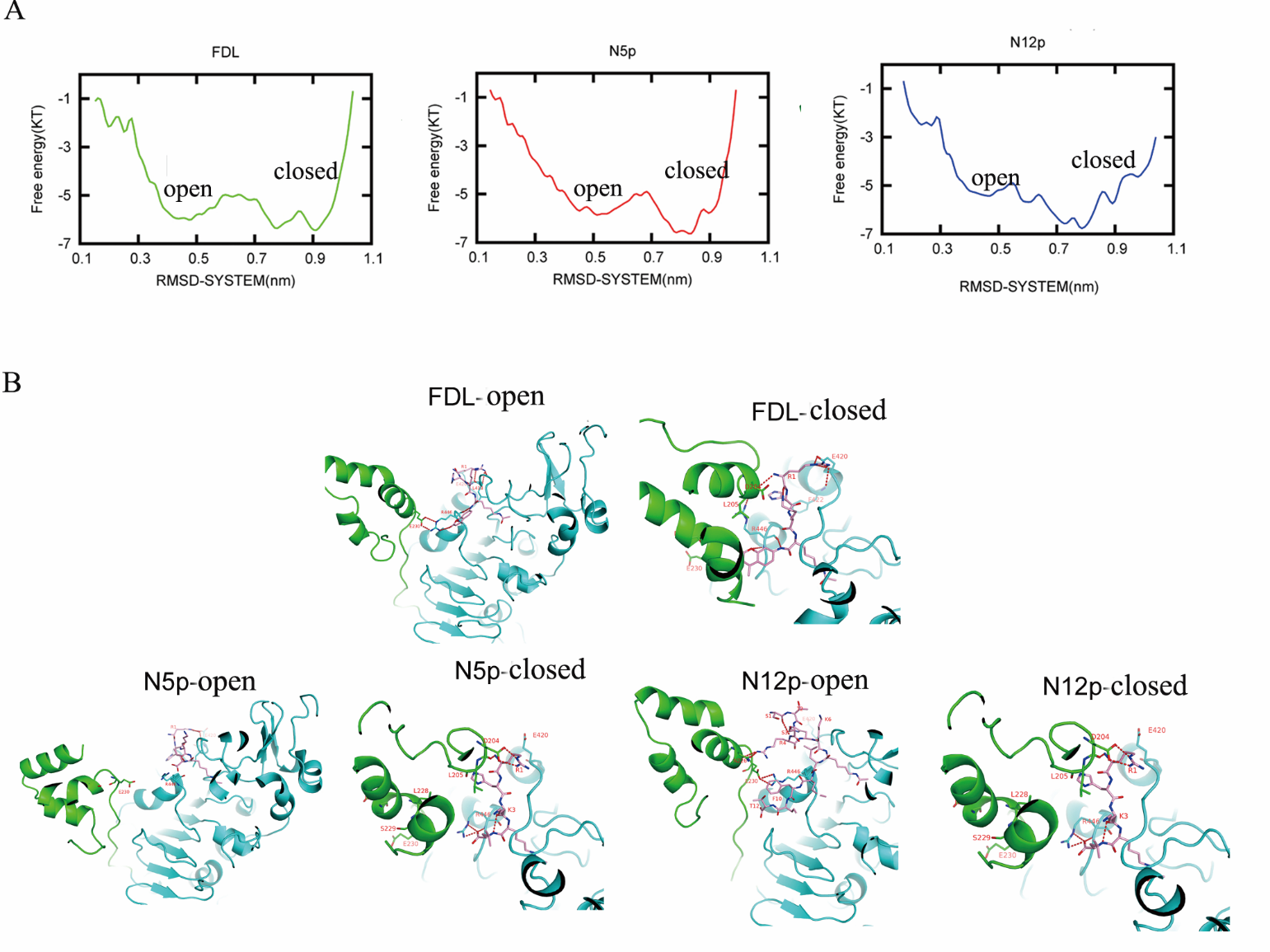


Figure S12. The one-dimensional landscape of the interaction between SIRT1 and p53. (A) The one-dimensional free energy landscape of the RMSD of SIRT1 and p53 peptides without STACs related to the open conformation (PDB:4zzj). (B) Conformations extracted from each state are shown.


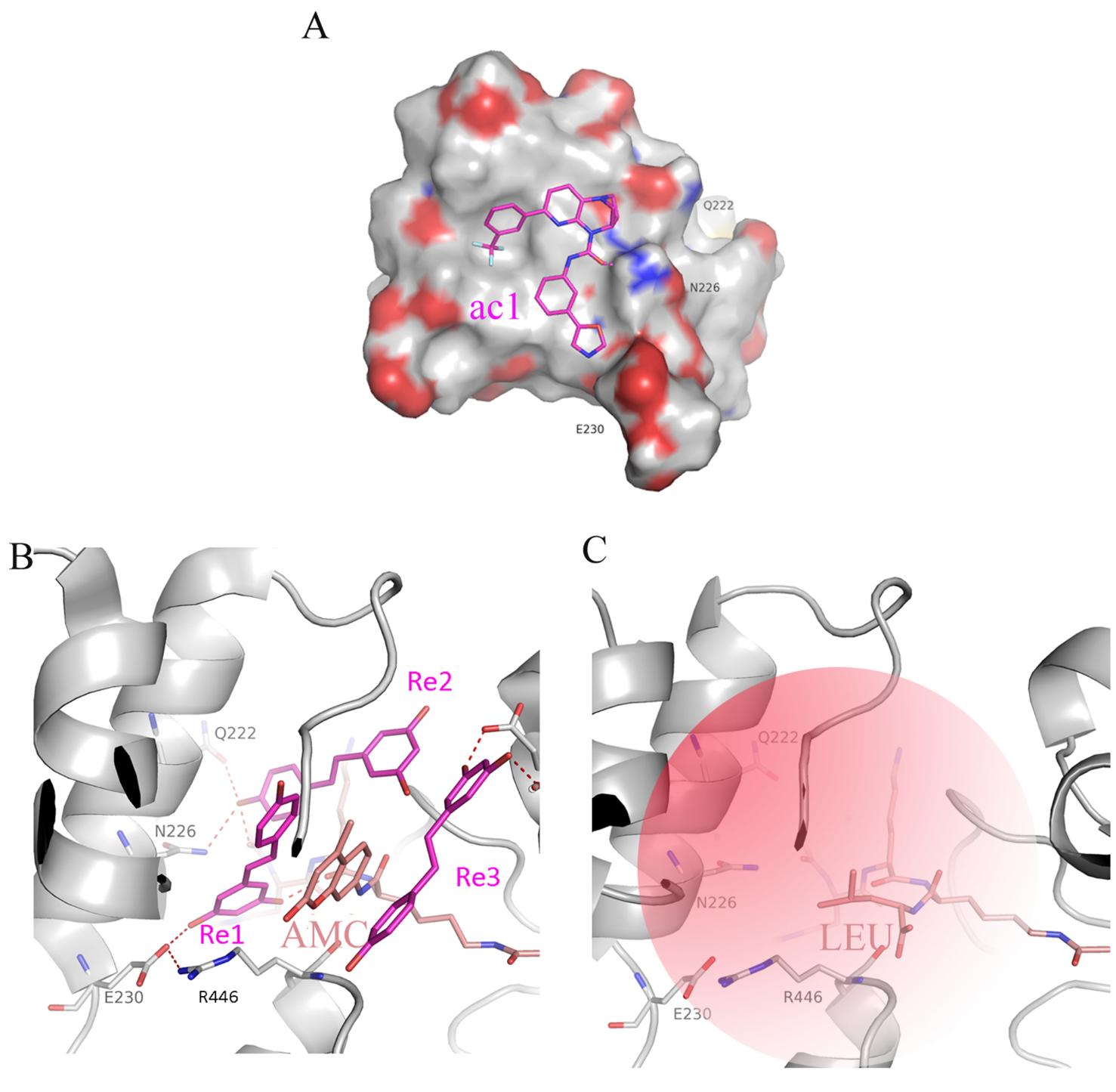


Figure S13. The design of HMD. (A-B) The binding model of NTD with ac1 and the active site of SIRT1-FDL-resveratol are shown. The ac1 and resveratrol were colored magenta in stick model. The FDL was colored salmon. Hydrogen bonds are shown by red dotted lines. In the surface model of NTD, the O, N and C atoms were colored by red, blue and gray, respectively. (C) The modified closed structure was used for HMD by replacing AMC group with a leucine and removing all resveratrols of SIRT1-FDL-resveratrol. The red sphere shadow represents the docking grid.


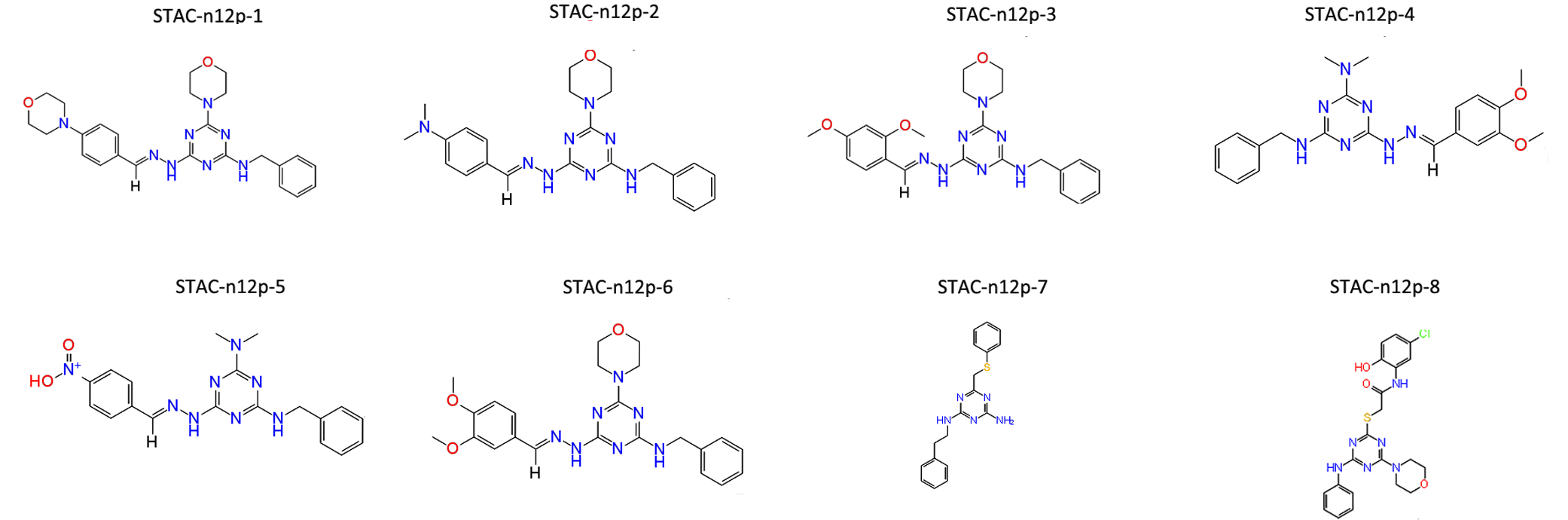


Figure S14. Structure of newfound STACs with *in vitro* activities.


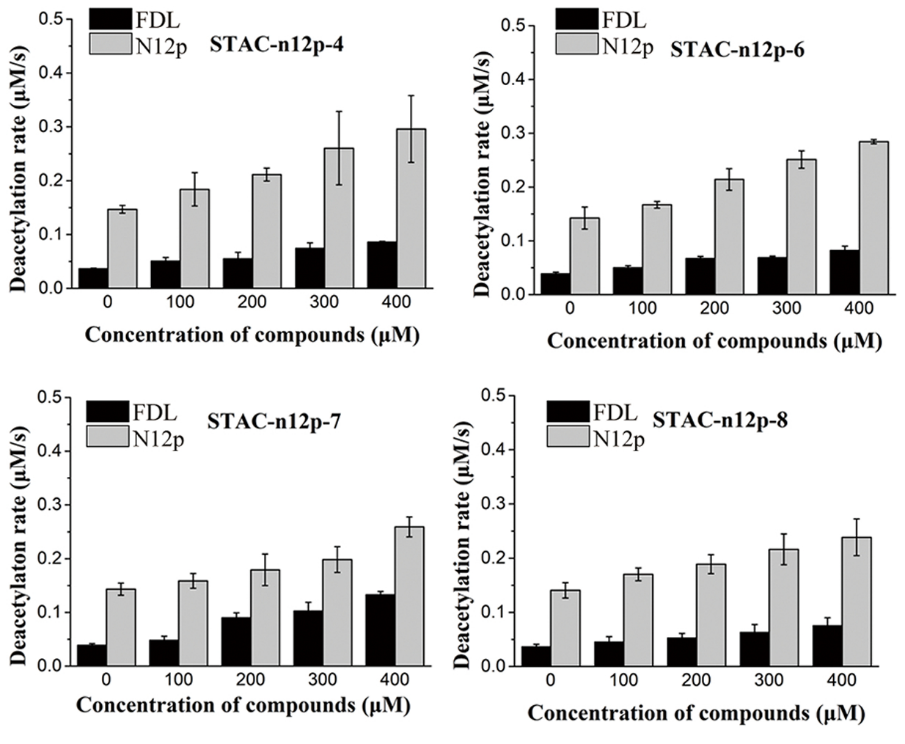


Figure S15. *In vitro* enzyme-coupled deacetylation (ECD) assay results of SIRT1 against FDL and N12p in the presence of various concentrations of compounds. The X axis indicates the concentrations of compounds, and the Y axis indicates the deacetylation rate of SIRT1 against substrate peptides.


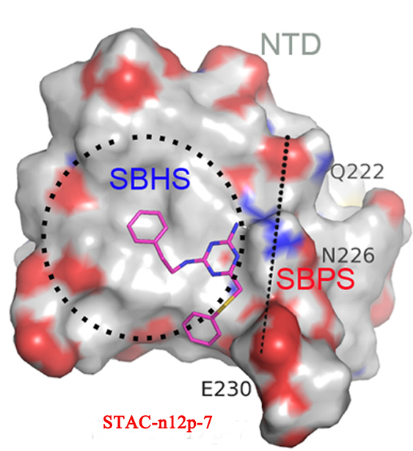


Figure S16. Surface binding model of SIRT1-NTD with compound STAC-n12p-7. The compound is shown by stick model in magenta. O, N, and C atoms in SIRT1 NTD are colored red, blue and gray, respectively. The SBHS is marked by dotted circle and the SBPS is marked by dotted line.


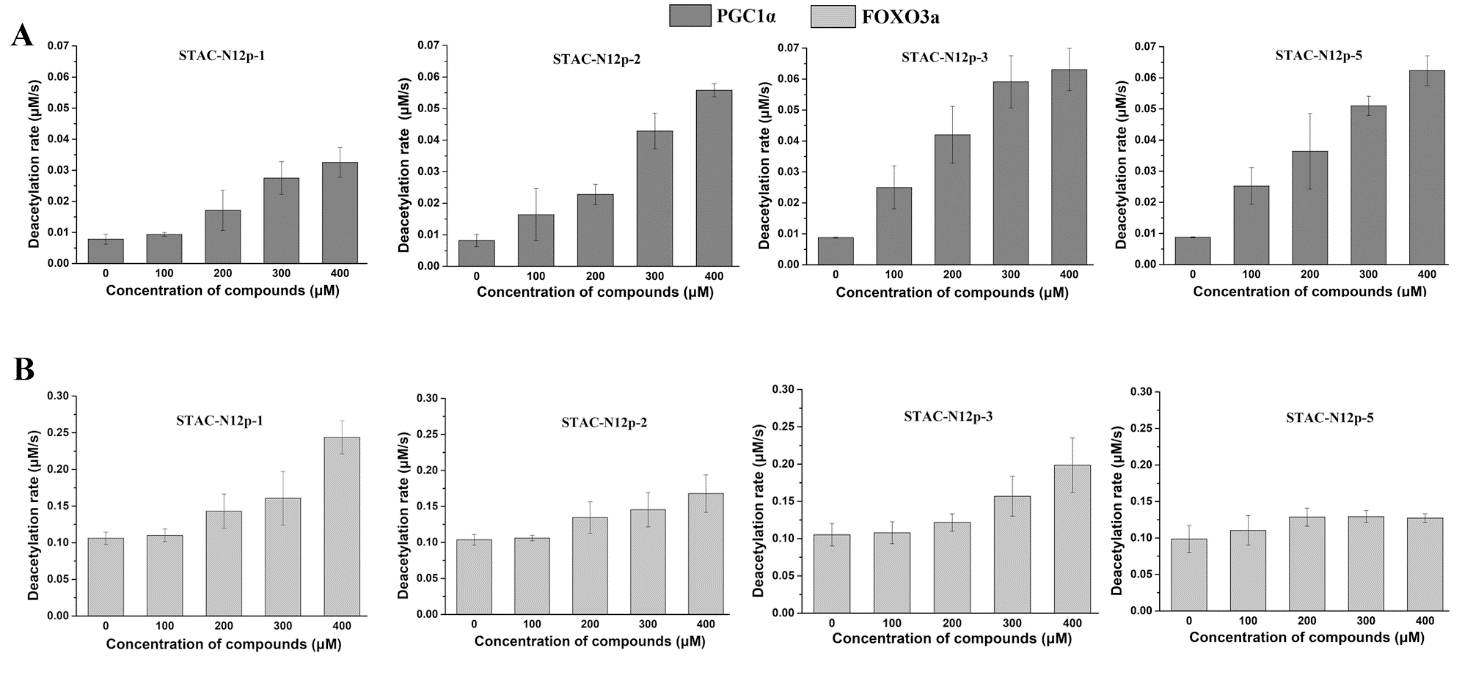


Figure S17. *In vitro* enzyme-coupled deacetylation (ECD) assay results of SIRT1 against PGC1α and FOXO3a in the presence of various concentrations of compounds.


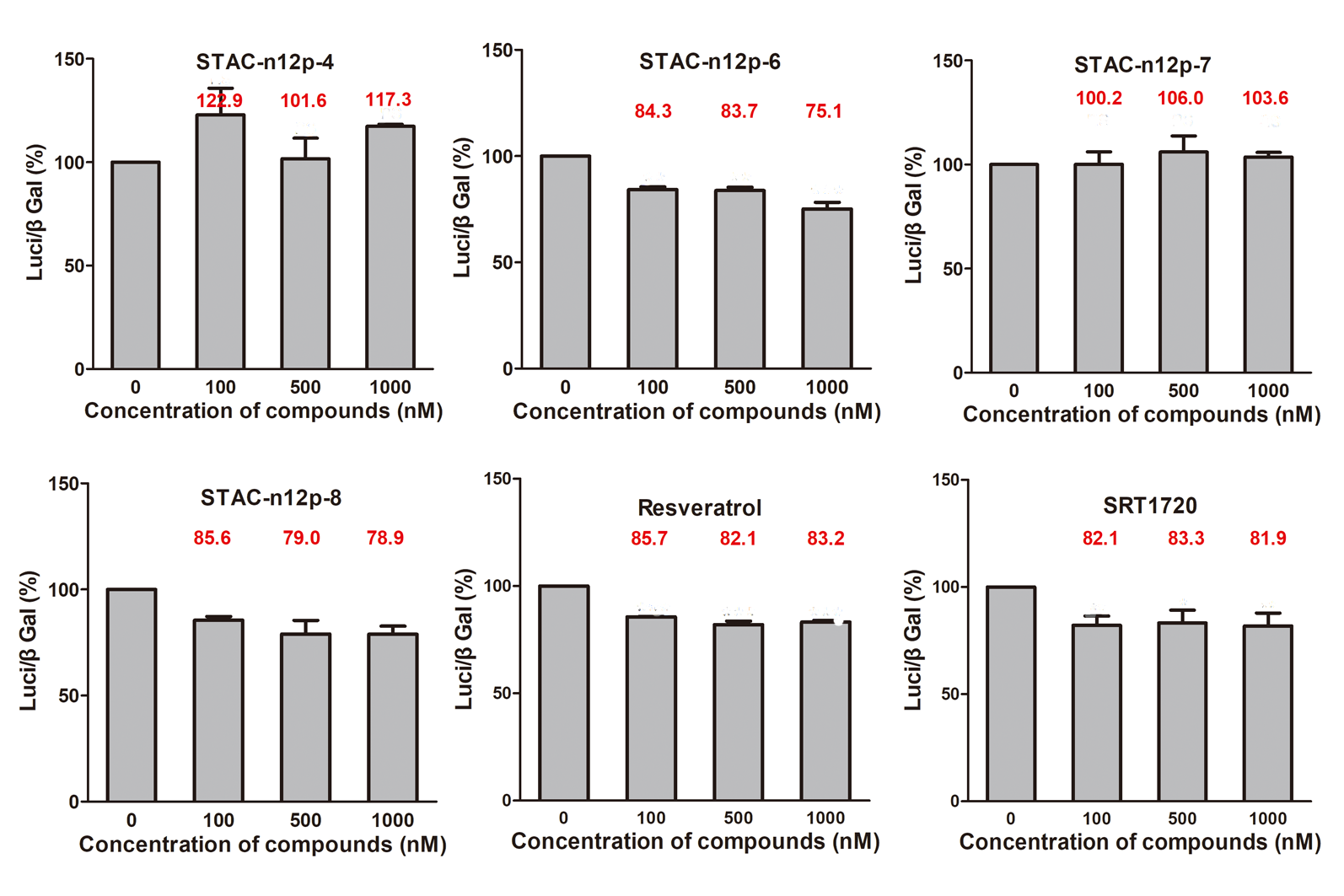


Figure S18. *In cell* activation of SIRT1 by newfound STACs examined by deacetylation assays in HEK293T cell. Reported STACS Resveratrol and SRT1720 were used as control. The X axis indicates the concentrations of compounds, and the Y axis shows reduction of luciferase activity indicating the activation activity of STACs.


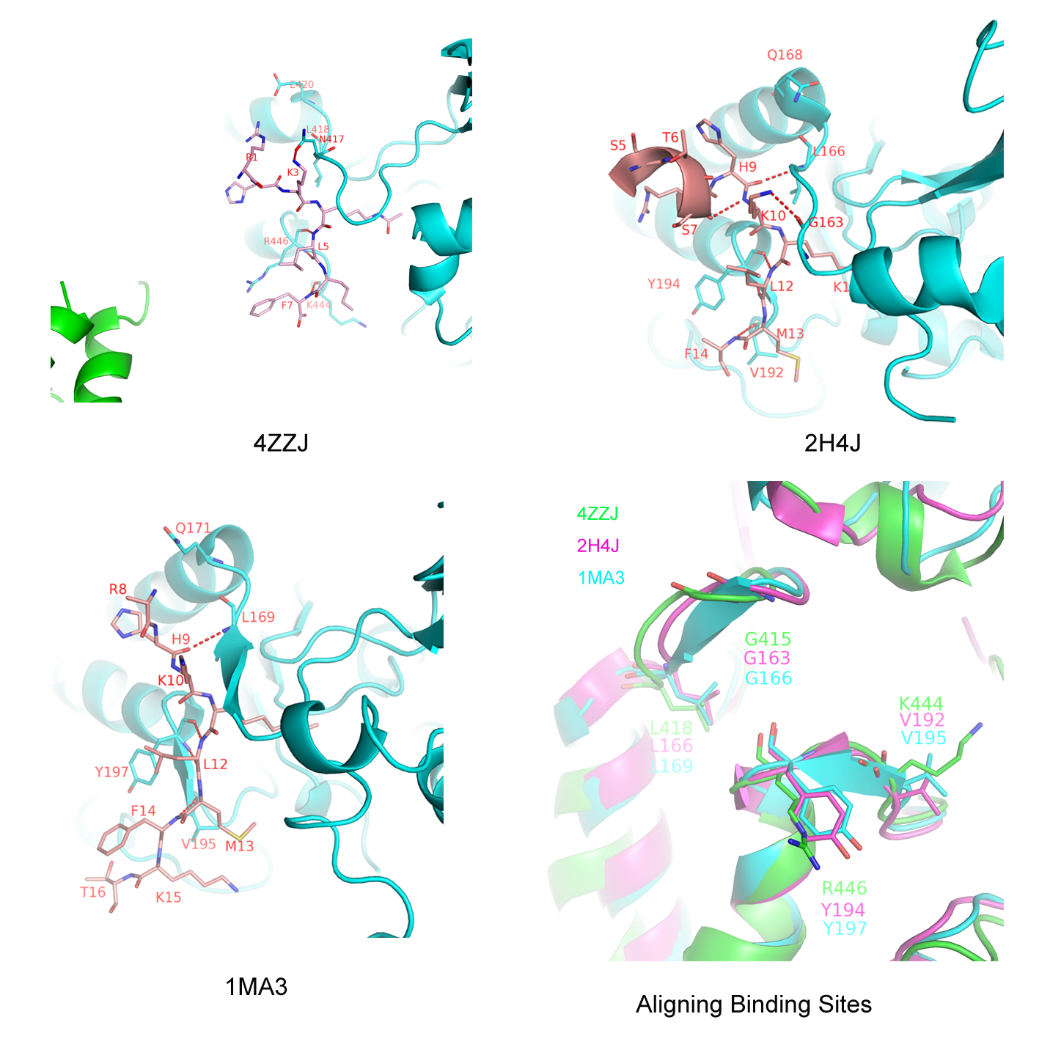


Figure S19. The interactions between SIRT1 and p53 peptide in different PDB structures. The p53 peptides and residues in the binding sites are shown by stick model. O and N atoms are colored red and blue, respectively. Hydrogen bonds are shown by red dotted lines. Three structures, PDB 4ZZJ, 2H4J and 1MA3 are overlapped and colored by green, magenta and cyan, respectively.


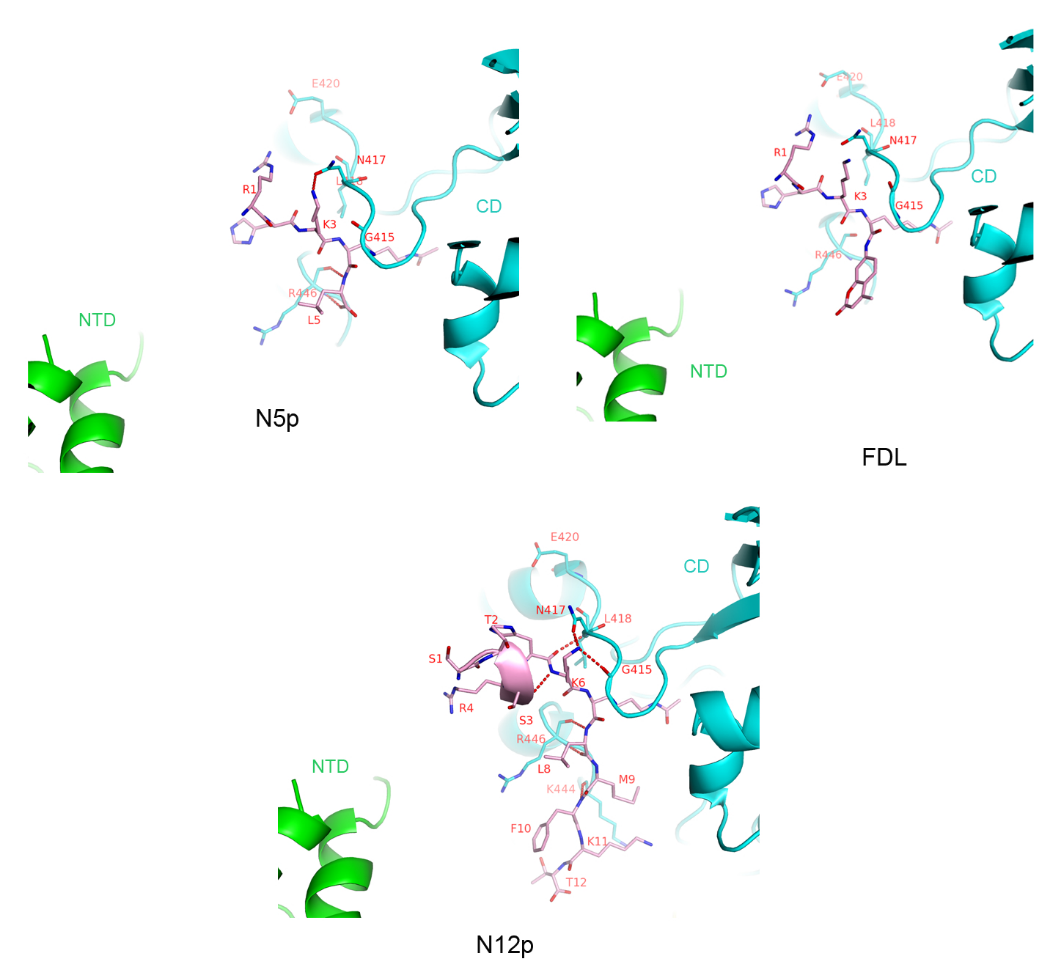


Figure S20. The interaction sites of p53 peptides including N5p, FDL and N12p on initial open structure of SIRT1. The p53 peptides and residues in the binding sites are shown by stick model. The CD domain is colored by cyan and the NTD domain is colored by green. Hydrogen bonds are shown by red dotted lines.


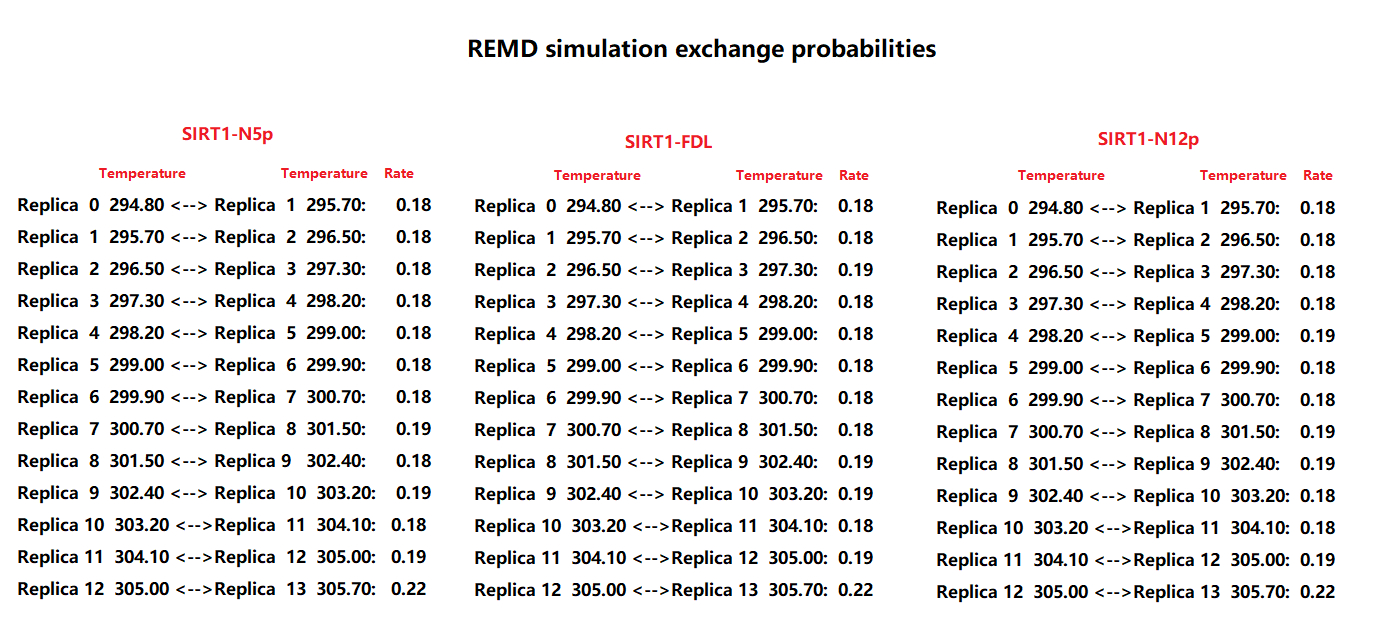


Figure S21. The exchange probabilities of REMD simulations. The REMD simulations were performed on three systems including SIRT1-N5p, -FDL, -N12p, and each system was performed by 14 replicas.


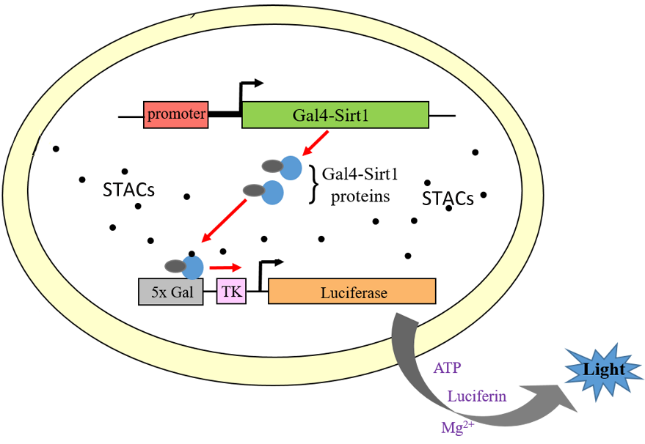


Figure S22. The schematic diagram of deacetylase assays of SIRT1 in cells.
